# Supplementary material for: A multi-disciplinary approach to identify spillover interfaces of bat coronaviruses to pig farms in Italy
Source: PLoS One. 2025 Oct 15;20(10):e0332117. doi: 10.1371/journal.pone.0332117 (PMC12527140; doi:10.1371/journal.pone.0332117)
Supplement: S1 Table — (DOCX) [file pone.0332117.s001.docx]

**Table S1. Descriptive summary of the variables used in the study with the relative metrics.**

|  | **PARAMETER NAME** | **DESCRIPTION** | **Data source** |
| --- | --- | --- | --- |
| Farm related | Farm area | Area of the farm, expressed in squared meters | Google Earth and ArcGIS Pro |
|  | Building age | Age of construction of the building; 0(NA), 1(OLD; > 10 years), 2(NEW; < 10 years) | Owner’s interview |
|  | Number of pigs | Number of pigs stabled in the farm | Owner’s interview |
|  | Sewage tank | Area of the tank, expressed in squared meters | Google Earth |
|  | Empty rooms | Presence of empty rooms in the farm; 0(NO), 1(YES) | Direct evaluation |
|  | Illumination | Presence of illumination sources; 1 (1 or less), 2 (2-5) | Direct evaluation |
|  | Irrigation canal | Presence of irrigation canals in the farm; 0(NO), 1(YES) | Direct evaluation |
|  | Noise | Presence of elevated and frequent noise due to sources such as airport, roads, railways; 0(NO), 1(YES) | Direct evaluation |
|  | Shutters | Presence of shutters in the farm; 0(NO), 1(YES) | Direct evaluation |
|  | Space behind gutters | Presence of space behind gutters; 0(NO), 1(YES) | Direct evaluation |
|  | Brocken bricks or crevices | Holes in masonry and walls; 0(NO), 1(YES) | Direct evaluation |
|  | Holed trees | Presence of hollow trees in the farm; 0(NO), 1(YES) | Direct evaluation |
|  | Opened doors/windows (animal sheds) | Presence of windows or doors allowing bats to enter animal sheds; 0(NO), 1(YES) | Direct evaluation |
|  | Opened doors/windows (feed storage) | Presence of windows or doors allowing bats to enter feed storage rooms; 0(NO), 1(YES) | Direct evaluation |
|  | Grills in air intake inlets | Presence of grills or nets at the air intake inlets | Direct evaluation |
|  | Presence of guano | Presence of guano indicating bat roosting; 0(NO), 1(YES) | Direct evaluation |
| Landscape related | Anthropogenic structures | Total area including buildings and other man-made structures inside the buffers, expressed in square meters. This area always included the actual farms | Google Earth and ArcGIS Pro |
|  | Agriculture environments | Total area devolved to agriculture inside the buffers, expressed in square meters | Google Earth and ArcGIS Pro |
|  | Wood | Total area of woods inside the buffers, expressed in square meters | Google Earth and ArcGIS Pro |
|  | Water bodies | Total area of water sources inside the buffers, expressed in square meters | Google Earth and ArcGIS Pro |
|  | Distance from the water | Distance from the first patch of water, expressed in meters | Google Earth and ArcGIS Pro |
|  | Distance from the wood | Distance from the first patch of wood, expressed in meters | Google Earth and ArcGIS Pro |
|  | Number of patches | Total number of patches (evaluation of the degree of landscape heterogeneity) | ArcGIS Pro |
