# Supplementary material for: A multi-disciplinary approach to identify spillover interfaces of bat coronaviruses to pig farms in Italy
Source: PLoS One. 2025 Oct 15;20(10):e0332117. doi: 10.1371/journal.pone.0332117 (PMC12527140; doi:10.1371/journal.pone.0332117)
Supplement: S2 Table — (DOCX) [file pone.0332117.s002.docx]

**Table S2. Descriptive summary of variables included in the analysis.**

|  |  |  | Mean | Median | Lower Quartile | Higher Quartile | Minimum | Maximum |
| --- | --- | --- | --- | --- | --- | --- | --- | --- |
| Response variables | | Activity (overall) | 53.34 | 17 | 9 | 42.75 | 0 | 497 |
|  |  | Activity (P. khulii only) | 32.69 | 12 | 5 | 33.5 | 0 | 162 |
|  |  | Richness | 2.57 | 2 | 1 | 4 | 0 | 7 |
| Predictors | Farm related | Farm area | 23138.97 | 17341 | 9076 | 40680 | 1215 | 65203 |
|  |  | Building age | 0.58 | 1 | 0 | 1 | 0 | 1 |
|  |  | Number of pigs | 3237.63 | 2500 | 300 | 5000 | 101 | 14315 |
|  |  | Size of sewage tank | 689.67 | 450 | 0 | 1619 | 0 | 1996 |
|  |  | Presence of empty rooms | 0.36 | 0 | 0 | 1 | 0 | 1 |
|  |  | Presence of illumination | 0.31 | 0 | 0 | 1 | 0 | 1 |
|  |  | Presence of irrigation canals | 0.43 | 0 | 0 | 1 | 0 | 1 |
|  |  | Presence of shutters | 0.27 | 0 | 0 | 1 | 0 | 1 |
|  |  | Presence of space behind gutters | 0.43 | 0 | 0 | 1 | 0 | 1 |
|  |  | Presence of holed trees | 0.26 | 0 | 0 | 0.75 | 0 | 1 |
|  | Landscape related | Anthropogenic structures (proportion) | 0.16 | 0.14 | 0.09 | 0.23 | 0 | 0.31 |
|  |  | Agriculture environments (proportion) | 0.76 | 0.8 | 0.73 | 0.86 | 0.35 | 0.91 |
|  |  | Wood (proportion) | 0.06 | 0 | 0 | 0.04 | 0 | 0.62 |
|  |  | Water bodies (proportion) | 0.01 | 0 | 0 | 0.01 | 0 | 0.15 |
|  |  | Distance from the water | 493.92 | 320 | 67.5 | 650 | 0 | 2500 |
|  |  | Distance from the wood | 350.66 | 332 | 148 | 383 | 0 | 820 |
|  |  | Number of patches | 8.55 | 6 | 4 | 11 | 3 | 21 |
