# Supplementary material for: A multi-disciplinary approach to identify spillover interfaces of bat coronaviruses to pig farms in Italy
Source: PLoS One. 2025 Oct 15;20(10):e0332117. doi: 10.1371/journal.pone.0332117 (PMC12527140; doi:10.1371/journal.pone.0332117)
Supplement: S3 Table — (DOCX) [file pone.0332117.s003.docx]

**Table S3. Description of the acoustic sample divided per farm.**

The Table reports i) the total bat activity expressed and number of bat passes per farm, determined over the number of recording days, ii) the total feeding activity - also called buzz-ratio - defined as the percentage of feeding buzzes over the total passes occurring in each farm, iii) the total social activity expressed as the percentage of social calls over the total passes occurring in each farm per recording night and iv) bat richness, referring to the number of bat species detected per farm.

| **FARM N°** | **Total Bat activity**  **(n passes/night)** | **Total Feeding activity (%)** | **Total Social activity (%)** | **Bat richness (n)** |
| --- | --- | --- | --- | --- |
| **1** | 14,6 | 4,00% | 2,86% | 2 |
| **2** | 16,6 | 0,00% | 3,89% | 2 |
| **3** | 9 | 3,70% | 0,00% | 2 |
| **4** | 15 | 0,00% | 0,00% | 3 |
| **5** | 64 | 0,00% | 0,00% | 4 |
| **6** | 12 | 0,00% | 0,00% | 2 |
| **7** | 8,16 | 0,00% | 0,00% | 1 |
| **8** | 5,75 | 0,00% | 4,35% | 6 |
| **9** | 15 | 0,00% | 2,73% | 5 |
| **10** | 36,8 | 4,90% | 9,31% | 3 |
| **11** | 44,4 | 5,14% | 6,54% | 3 |
| **12** | 13,3 | 0,00% | 5,08% | 5 |
| **13** | 211,63 | 0,00% | 0,00068% | 4 |
| **14** | 171,3 | 0,00% | 4,82% | 5 |
