# Supplementary material for: A multi-disciplinary approach to identify spillover interfaces of bat coronaviruses to pig farms in Italy
Source: PLoS One. 2025 Oct 15;20(10):e0332117. doi: 10.1371/journal.pone.0332117 (PMC12527140; doi:10.1371/journal.pone.0332117)
Supplement: S4 Table — (DOCX) [file pone.0332117.s004.docx]

**Table S4. Description of the acoustic sample divided per farm and per species.**

The activity is expressed as the number of species-specific passes per farm determined over the number of recording days per farm; Occurrence represents the percentage of species-specific passes determined over the total number of passes per farm; the feeding activity is the percentage of species-specific feeding buzzes over the total number of feeding buzzes per farm; Social activity is the percentage of species-specific social calls over the total number of social calls per farm.

| \| **FARM N°** \| **Species** \| **Activity (n passes/nights)** \| **Occurrence (%)** \| **Feeding activity (%)** \| **Social activity (%)** \| \| --- \| --- \| --- \| --- \| --- \| --- \| \| **1** \| *Pipistrellus kuhlii* \| 12,4 \| 87,00% \| 33,00% \| 50,00% \| \|  \| *Pipistrellus pipistrellus* \| 9,8 \| 12,67% \| 66,00% \| 50,00% \| \|  \| *Hypsugo savii* \| 0 \| 0,00% \| 0,00% \| 0,00% \| \|  \| *Eptesicus serotinus* \| 0 \| 0,00% \| 0,00% \| 0,00% \| \|  \| *Nyctalus leislerii* \| 0 \| 0,00% \| 0,00% \| 0,00% \| \|  \| *Rhinolophus ferrumequinum* \| 0 \| 0,00% \| 0,00% \| 0,00% \| \|  \| *Rhinolophus hipposideros* \| 0 \| 0,00% \| 0,00% \| 0,00% \| \|  \| *Myotis sp.* \| 0 \| 0,00% \| 0,00% \| 0,00% \| \| **2** \| *Pipistrellus kuhlii* \| 10,8 \| 70,00% \| 0,00% \| 33,00% \| \|  \| *Pipistrellus pipistrellus* \| 4,6 \| 30,00% \| 0,00% \| 66,00% \| \|  \| *Hypsugo savii* \| 0 \| 0,00% \| 0,00% \| 0,00% \| \|  \| *Eptesicus serotinus* \| 0 \| 0,00% \| 0,00% \| 0,00% \| \|  \| *Nyctalus leislerii* \| 0 \| 0,00% \| 0,00% \| 0,00% \| \|  \| *Rhinolophus ferrumequinum* \| 0 \| 0,00% \| 0,00% \| 0,00% \| \|  \| *Rhinolophus hipposideros* \| 0 \| 0,00% \| 0,00% \| 0,00% \| \|  \| *Myotis sp.* \| 0 \| 0,00% \| 0,00% \| 0,00% \| \| **3** \| *Pipistrellus kuhlii* \| 8 \| 88,88% \| 100,00% \| 0,00% \| \|  \| *Pipistrellus pipistrellus* \| 1 \| 11,11% \| 0,00% \| 0,00% \| \|  \| *Hypsugo savii* \| 0 \| 0,00% \| 0,00% \| 0,00% \| \|  \| *Eptesicus serotinus* \| 0 \| 0,00% \| 0,00% \| 0,00% \| \|  \| *Nyctalus leislerii* \| 0 \| 0,00% \| 0,00% \| 0,00% \| \|  \| *Rhinolophus ferrumequinum* \| 0 \| 0,00% \| 0,00% \| 0,00% \| \|  \| *Rhinolophus hipposideros* \| 0 \| 0,00% \| 0,00% \| 0,00% \| \|  \| *Myotis sp.* \| 0 \| 0,00% \| 0,00% \| 0,00% \| \| **FARM N°** \| **Species** \| **Activity (n passes/nights)** \| **Occurrence (%)** \| **Feeding activity (%)** \| **Social activity (%)** \| \| **4** \| *Pipistrellus kuhlii* \| 12,8 \| 88,88% \| 0,00% \| 0,00% \| \|  \| *Pipistrellus pipistrellus* \| 1 \| 6,94% \| 0,00% \| 0,00% \| \|  \| *Hypsugo savii* \| 0,6 \| 4,16% \| 0,00% \| 0,00% \| \|  \| *Eptesicus serotinus* \| 0 \| 0,00% \| 0,00% \| 0,00% \| \|  \| *Nyctalus leislerii* \| 0 \| 0,00% \| 0,00% \| 0,00% \| \|  \| *Rhinolophus ferrumequinum* \| 0 \| 0,00% \| 0,00% \| 0,00% \| \|  \| *Rhinolophus hipposideros* \| 0 \| 0,00% \| 0,00% \| 0,00% \| \|  \| *Myotis sp.* \| 0 \| 0,00% \| 0,00% \| 0,00% \| \| **5** \| *Pipistrellus kuhlii* \| 55,66 \| 91,75% \| 0,00% \| 0,00% \| \|  \| *Pipistrellus pipistrellus* \| 3,3 \| 5,49% \| 0,00% \| 0,00% \| \|  \| *Hypsugo savii* \| 0,66 \| 1,09% \| 0,00% \| 0,00% \| \|  \| *Eptesicus serotinus* \| 1 \| 1,64% \| 0,00% \| 0,00% \| \|  \| *Nyctalus leislerii* \| 0 \| 0,00% \| 0,00% \| 0,00% \| \|  \| *Rhinolophus ferrumequinum* \| 0 \| 0,00% \| 0,00% \| 0,00% \| \|  \| *Rhinolophus hipposideros* \| 0 \| 0,00% \| 0,00% \| 0,00% \| \|  \| *Myotis sp.* \| 0 \| 0,00% \| 0,00% \| 0,00% \| \| **6** \| *Pipistrellus kuhlii* \| 10,6 \| 86,88% \| 0,00% \| 0,00% \| \|  \| *Pipistrellus pipistrellus* \| 1,6 \| 13,11% \| 0,00% \| 0,00% \| \|  \| *Hypsugo savii* \| 0 \| 0,00% \| 0,00% \| 0,00% \| \|  \| *Eptesicus serotinus* \| 0 \| 0,00% \| 0,00% \| 0,00% \| \|  \| *Nyctalus leislerii* \| 0 \| 0,00% \| 0,00% \| 0,00% \| \|  \| *Rhinolophus ferrumequinum* \| 0 \| 0,00% \| 0,00% \| 0,00% \| \|  \| *Rhinolophus hipposideros* \| 0 \| 0,00% \| 0,00% \| 0,00% \| \|  \| *Myotis sp.* \| 0 \| 0,00% \| 0,00% \| 0,00% \| \| **7** \| *Pipistrellus kuhlii* \| 8,16 \| 100,00% \| 0,00% \| 0,00% \| \|  \| *Pipistrellus pipistrellus* \| 0 \| 0,00% \| 0,00% \| 0,00% \| \|  \| *Hypsugo savii* \| 0 \| 0,00% \| 0,00% \| 0,00% \| \|  \| *Eptesicus serotinus* \| 0 \| 0,00% \| 0,00% \| 0,00% \| \|  \| *Nyctalus leislerii* \| 0 \| 0,00% \| 0,00% \| 0,00% \| \|  \| *Rhinolophus ferrumequinum* \| 0 \| 0,00% \| 0,00% \| 0,00% \| \|  \| *Rhinolophus hipposideros* \| 0 \| 0,00% \| 0,00% \| 0,00% \| \|  \| *Myotis sp.* \| 0 \| 0,00% \| 0,00% \| 0,00% \| \| **FARM N°** \| **Species** \| **Activity (n passes/nights)** \| **Occurrence (%)** \| **Feeding activity (%)** \| **Social activity (%)** \| \| **8** \| *Pipistrellus kuhlii* \| 3,42 \| 55,81% \| 0,00% \| 50,00% \| \|  \| *Pipistrellus pipistrellus* \| 1,57 \| 25,58% \| 0,00% \| 50,00% \| \|  \| *Hypsugo savii* \| 0,28 \| 4,65% \| 0,00% \| 0,00% \| \|  \| *Eptesicus serotinus* \| 0 \| 0,00% \| 0,00% \| 0,00% \| \|  \| *Nyctalus leislerii* \| 0 \| 0,00% \| 0,00% \| 0,00% \| \|  \| *Rhinolophus ferrumequinum* \| 0,28 \| 4,65% \| 0,00% \| 0,00% \| \|  \| *Rhinolophus hipposideros* \| 0,28 \| 4,65% \| 0,00% \| 0,00% \| \|  \| *Myotis sp.* \| 0,28 \| 4,65% \| 0,00% \| 0,00% \| \| **9** \| *Pipistrellus kuhlii* \| 6,6 \| 45,20% \| 0,00% \| 50,00% \| \|  \| *Pipistrellus pipistrellus* \| 1,6 \| 10,95% \| 0,00% \| 50,00% \| \|  \| *Hypsugo savii* \| 2,8 \| 19,17% \| 0,00% \| 0,00% \| \|  \| *Eptesicus serotinus* \| 2,4 \| 16,43% \| 0,00% \| 0,00% \| \|  \| *Nyctalus leislerii* \| 1,4 \| 8,22% \| 0,00% \| 0,00% \| \|  \| *Rhinolophus ferrumequinum* \| 0 \| 0,00% \| 0,00% \| 0,00% \| \|  \| *Rhinolophus hipposideros* \| 0 \| 0,00% \| 0,00% \| 0,00% \| \|  \| *Myotis sp.* \| 0 \| 0,00% \| 0,00% \| 0,00% \| \| **10** \| *Pipistrellus kuhlii* \| 26,83 \| 78,92% \| 100,00% \| 100,00% \| \|  \| *Pipistrellus pipistrellus* \| 7 \| 20,58% \| 0,00% \| 0,00% \| \|  \| *Hypsugo savii* \| 0,16 \| 0,49% \| 0,00% \| 0,00% \| \|  \| *Eptesicus serotinus* \| 0 \| 0,00% \| 0,00% \| 0,00% \| \|  \| *Nyctalus leislerii* \| 0 \| 0,00% \| 0,00% \| 0,00% \| \|  \| *Rhinolophus ferrumequinum* \| 0 \| 0,00% \| 0,00% \| 0,00% \| \|  \| *Rhinolophus hipposideros* \| 0 \| 0,00% \| 0,00% \| 0,00% \| \|  \| *Myotis sp.* \| 0 \| 0,00% \| 0,00% \| 0,00% \| \| **11** \| *Pipistrellus kuhlii* \| 35,8 \| 83,64% \| 100,00% \| 92,85% \| \|  \| *Pipistrellus pipistrellus* \| 6,6 \| 15,42% \| 0,00% \| 7,00% \| \|  \| *Hypsugo savii* \| 0,4 \| 0,93% \| 0,00% \| 0,00% \| \|  \| *Eptesicus serotinus* \| 0 \| 0,00% \| 0,00% \| 0,00% \| \|  \| *Nyctalus leislerii* \| 0 \| 0,00% \| 0,00% \| 0,00% \| \|  \| *Rhinolophus ferrumequinum* \| 0 \| 0,00% \| 0,00% \| 0,00% \| \|  \| *Rhinolophus hipposideros* \| 0 \| 0,00% \| 0,00% \| 0,00% \| \|  \| *Myotis sp.* \| 0 \| 0,00% \| 0,00% \| 0,00% \|  \| **FARM N°** \| **Species** \| **Activity (n passes/nights)** \| **Occurrence (%)** \| **Feeding activity (%)** \| **Social activity (%)** \| \| --- \| --- \| --- \| --- \| --- \| --- \| \| **12** \| *Pipistrellus kuhlii* \| 5,83 \| 59,32% \| 0,00% \| 0,00% \| \|  \| *Pipistrellus pipistrellus* \| 0,33 \| 3,39% \| 0,00% \| 0,00% \| \|  \| *Hypsugo savii* \| 2 \| 20,33% \| 0,00% \| 100,00% \| \|  \| *Eptesicus serotinus* \| 1,66 \| 16,95% \| 0,00% \| 0,00% \| \|  \| *Nyctalus leislerii* \| 0 \| 0,00% \| 0,00% \| 0,00% \| \|  \| *Rhinolophus ferrumequinum* \| 0 \| 0,00% \| 0,00% \| 0,00% \| \|  \| *Rhinolophus hipposideros* \| 0 \| 0,00% \| 0,00% \| 0,00% \| \|  \| *Myotis sp.* \| 0,5 \| 5,08% \| 0,00% \| 0,00% \| \| **13** \| *Pipistrellus kuhlii* \| 96,54 \| 72,24% \| 0,00% \| 0,00% \| \|  \| *Pipistrellus pipistrellus* \| 34,63 \| 25,91% \| 0,00% \| 0,00% \| \|  \| *Hypsugo savii* \| 1,54 \| 1,15% \| 0,00% \| 100,00% \| \|  \| *Eptesicus serotinus* \| 0,9 \| 0,68% \| 0,00% \| 0,00% \| \|  \| *Nyctalus leislerii* \| 0 \| 0,00% \| 0,00% \| 0,00% \| \|  \| *Rhinolophus ferrumequinum* \| 0 \| 0,00% \| 0,00% \| 0,00% \| \|  \| *Rhinolophus hipposideros* \| 0 \| 0,00% \| 0,00% \| 0,00% \| \|  \| *Myotis sp.* \| 0 \| 0,00% \| 0,00% \| 0,00% \| \| **14** \| *Pipistrellus kuhlii* \| 136,6 \| 80,70% \| 0,00% \| 75,51% \| \|  \| *Pipistrellus pipistrellus* \| 22,83 \| 13,48% \| 0,00% \| 4,08% \| \|  \| *Hypsugo savii* \| 6,8 \| 4,03% \| 0,00% \| 20,41% \| \|  \| *Eptesicus serotinus* \| 3 \| 0,30% \| 0,00% \| 0,00% \| \|  \| *Nyctalus leislerii* \| 0 \| 0,00% \| 0,00% \| 0,00% \| \|  \| *Rhinolophus ferrumequinum* \| 0 \| 0,00% \| 0,00% \| 0,00% \| \|  \| *Rhinolophus hipposideros* \| 0 \| 0,00% \| 0,00% \| 0,00% \| \|  \| *Myotis sp.* \| 0,66 \| 0,39% \| 0,00% \| 0,00% \| |
| --- | --- | --- | --- | --- | --- | --- | --- | --- | --- | --- | --- | --- | --- | --- | --- | --- | --- | --- | --- | --- | --- | --- | --- | --- | --- | --- | --- | --- | --- | --- | --- | --- | --- | --- | --- | --- | --- | --- | --- | --- | --- | --- | --- | --- | --- | --- | --- | --- | --- | --- | --- | --- | --- | --- | --- | --- | --- | --- | --- | --- | --- | --- | --- | --- | --- | --- | --- | --- | --- | --- | --- | --- | --- | --- | --- | --- | --- | --- | --- | --- | --- | --- | --- | --- | --- | --- | --- | --- | --- | --- | --- | --- | --- | --- | --- | --- | --- | --- | --- | --- | --- | --- | --- | --- | --- | --- | --- | --- | --- | --- | --- | --- | --- | --- | --- | --- | --- | --- | --- | --- | --- | --- | --- | --- | --- | --- | --- | --- | --- | --- | --- | --- | --- | --- | --- | --- | --- | --- | --- | --- | --- | --- | --- | --- | --- | --- | --- | --- | --- | --- | --- | --- | --- | --- | --- | --- | --- | --- | --- | --- | --- | --- | --- | --- | --- | --- | --- | --- | --- | --- | --- | --- | --- | --- | --- | --- | --- | --- | --- | --- | --- | --- | --- | --- | --- | --- | --- | --- | --- | --- | --- | --- | --- | --- | --- | --- | --- | --- | --- | --- | --- | --- | --- | --- | --- | --- | --- | --- | --- | --- | --- | --- | --- | --- | --- | --- | --- | --- | --- | --- | --- | --- | --- | --- | --- | --- | --- | --- | --- | --- | --- | --- | --- | --- | --- | --- | --- | --- | --- | --- | --- | --- | --- | --- | --- | --- | --- | --- | --- | --- | --- | --- | --- | --- | --- | --- | --- | --- | --- | --- | --- | --- | --- | --- | --- | --- | --- | --- | --- | --- | --- | --- | --- | --- | --- | --- | --- | --- | --- | --- | --- | --- | --- | --- | --- | --- | --- | --- | --- | --- | --- | --- | --- | --- | --- | --- | --- | --- | --- | --- | --- | --- | --- | --- | --- | --- | --- | --- | --- | --- | --- | --- | --- | --- | --- | --- | --- | --- | --- | --- | --- | --- | --- | --- | --- | --- | --- | --- | --- | --- | --- | --- | --- | --- | --- | --- | --- | --- | --- | --- | --- | --- | --- | --- | --- | --- | --- | --- | --- | --- | --- | --- | --- | --- | --- | --- | --- | --- | --- | --- | --- | --- | --- | --- | --- | --- | --- | --- | --- | --- | --- | --- | --- | --- | --- | --- | --- | --- | --- | --- | --- | --- | --- | --- | --- | --- | --- | --- | --- | --- | --- | --- | --- | --- | --- | --- | --- | --- | --- | --- | --- | --- | --- | --- | --- | --- | --- | --- | --- | --- | --- | --- | --- | --- | --- | --- | --- | --- | --- | --- | --- | --- | --- | --- | --- | --- | --- | --- | --- | --- | --- | --- | --- | --- | --- | --- | --- | --- | --- | --- | --- | --- | --- | --- | --- | --- | --- | --- | --- | --- | --- | --- | --- | --- | --- | --- | --- | --- | --- | --- | --- | --- | --- | --- | --- | --- | --- | --- | --- | --- | --- | --- | --- | --- | --- | --- | --- | --- | --- | --- | --- | --- | --- | --- | --- | --- | --- | --- | --- | --- | --- | --- | --- | --- | --- | --- | --- | --- | --- | --- | --- | --- | --- | --- | --- | --- | --- | --- | --- | --- | --- | --- | --- | --- | --- | --- | --- | --- | --- | --- | --- | --- | --- | --- | --- | --- | --- | --- | --- | --- | --- | --- | --- | --- | --- | --- | --- | --- | --- | --- | --- | --- | --- | --- | --- | --- | --- | --- | --- | --- | --- | --- | --- | --- | --- | --- | --- | --- | --- | --- | --- | --- | --- | --- | --- | --- | --- | --- | --- | --- | --- | --- | --- | --- | --- | --- | --- | --- | --- | --- | --- | --- | --- | --- | --- | --- | --- | --- | --- | --- | --- | --- | --- | --- | --- | --- | --- | --- | --- | --- | --- | --- | --- | --- | --- | --- | --- | --- | --- | --- | --- | --- | --- | --- | --- | --- | --- | --- | --- | --- | --- | --- | --- | --- | --- | --- | --- | --- | --- | --- | --- | --- | --- | --- | --- | --- | --- | --- | --- | --- | --- | --- | --- | --- | --- | --- | --- | --- | --- | --- | --- | --- | --- | --- | --- | --- | --- | --- | --- | --- | --- | --- | --- | --- | --- | --- | --- | --- | --- | --- | --- | --- | --- | --- | --- | --- | --- | --- | --- | --- | --- | --- | --- | --- | --- | --- | --- | --- | --- | --- | --- | --- | --- | --- | --- | --- |

|  |
| --- |
