# Supplementary material for: A multi-disciplinary approach to identify spillover interfaces of bat coronaviruses to pig farms in Italy
Source: PLoS One. 2025 Oct 15;20(10):e0332117. doi: 10.1371/journal.pone.0332117 (PMC12527140; doi:10.1371/journal.pone.0332117)
Supplement: S5 Table — (DOCX) [file pone.0332117.s005.docx]

**Table S5. Descriptive summary of the results of the landscape analysis, divided per farm.**

|  | **Farm number** | **1** | **2** | **3** | **4** | **5** | **6** | **7** | **8** | **9** | **10** | **11** | **12** | **13** | **14** |
| --- | --- | --- | --- | --- | --- | --- | --- | --- | --- | --- | --- | --- | --- | --- | --- |
| **Farm related** | farm area (m^2^) | 20627 | 12913 | 12157 | 9076 | 14591 | 9277 | 65203 | 24899 | 19651 | 40680 | 40680 | 54140 | 51466 | 13332 |
|  | building ≥50 years old or new | old | - | old | old | new | - | old-new | old | new | new | new | new | new | old |
|  | number of pigs | 5134 | 2160 | 101 | 1704 | 2494 | 110 | 7940 | 300 | 150 | 5000 | 5000 | 3500 | 8000 | 2500 |
|  | sewage tanks | yes | no | yes | yes | yes | no | no | yes | no | yes | yes | no | yes | yes |
|  | sewage tanks area (m^2^) | 488 | - | 806 | 750 | 228 | - | - | 450 | - | 1758 | 1758 | 0 | 1619 | 1792 |
|  | possible loud noises (i.e. highway, aeroport) | no | no | no | no | no | no | no | no | no | no | no | yes | yes | no |
|  | empty unused rooms | no | no | no | no | no | no | yes | yes | no | no | no | yes | no | yes |
|  | Illumination (n of light sources ≤1 (poor), 2-5 (medium), > (high) | medium | poor | medium | poor | poor | poor | poor | poor | medium | poor | poor | medium | poor | poor |
|  | irrigation canals | no | yes | yes | no | yes | no | no | no | yes | yes | yes | no | no | yes |
|  | shutters | no | no | yes | yes | no | no | yes | no | no | no | no | no | no | yes |
|  | space behind gutters | yes | no | no | yes | no | no | no | yes | no | yes | yes | no | no | yes |
|  | holed briks | yes | yes | no | no | yes | no | no | yes | no | no | no | no | no | yes |
|  | holed trees | no | no | yes | no | no | yes | no | no | no | no | no | no | no | yes |
|  | open doors/window to animal shelters | yes | yes | yes | yes | yes | yes | yes | yes | yes | yes | yes | no | no | yes |
|  | open doors/window to feed storage rooms | no | no | no | - | no | yes | no | - | yes | yes | yes | yes | no | yes |
|  | presence of nets/grids in windows | no | no | no | no | yes | no | no | no | yes | no | no | no | no | no |
|  | presence of guano | no | no | no | yes | no | no | no | no | no | yes | yes | no | no | no |
| **Landscape related** | Anthropogenic structures (m^2^) | 66816 | 87124 | 42199 | 78498 | 19417 | 994.1 | 65203.9 | 24899.2 | 66634 | 40680 | 40680 | 40188.4 | 36176 | 23264.8 |
|  | Agriculture environments (m^2^) | 208918 | 97404 | 230347 | 203790 | 239175 | 107922.3 | 216317.2 | 259613.7 | 215780 | 240710 | 240710 | 242520.3 | 196644 | 259206.5 |
|  | Wood (m^2^) | 9729 | 56130 | 10516 | 0 | 21748 | 177813.5 | 0 | 0 | 0 | 0 | 0 | 0 | 39515 | 3505.9 |
|  | Water bodies (m^2^) | 2182 | 41288 | 3756 | 767 | 9726 | 0 | 0 | 0 | 3839 | 0 | 0 | 0 | 1741 | 0 |
|  | Distance from the water (m) | 220 | 146 | 7 | 147 | 26 | 2500 | 1078 | 320 | 55 | 478 | 478 | 650 | 10 | 557 |
|  | Distance from the wood (m) | 148 | 160 | 39 | 340 | 160 | 0 | 356 | 820 | 383 | 332 | 332 | 798 | 20 | 198 |
|  | Number of patches | 13 | 19 | 10 | 11 | 12 | 7 | 3 | 3 | 6 | 6 | 6 | 4 | 21 | 6 |
