# Supplementary material for: A multi-disciplinary approach to identify spillover interfaces of bat coronaviruses to pig farms in Italy
Source: PLoS One. 2025 Oct 15;20(10):e0332117. doi: 10.1371/journal.pone.0332117 (PMC12527140; doi:10.1371/journal.pone.0332117)
Supplement: S7 Table — (DOCX) [file pone.0332117.s007.docx]

**Table S7. This table presents equivalent results as the ones reported in Fig 3 in the main text. However, here up to 4 predictors have been included (instead of 3 in the main text).**

Summary of model-averaging coefficient when predicting either ‘overall activity’, ‘*P. kuhlii* activity’, or bat species ‘richness’. Coefficients, 95% Confidence Intervals (CI) and Relative importance (Ri) associated with each variable considered. When no estimate is provided, the variable was excluded from the analysis based on the preliminary univariate regression. Blue (red) shading indicates positive (negative) relationships, while darker shading indicates estimate for which the lower (upper) 95%CI bound did not overlap with 0 (i.e. this can be interpreted as a statistically significant impact under an hypothesis-testing framework).

|  | Variable | Overall activity  Coefficient (95%CI) ; Ri | P.k. only Activity  Coefficient (95%CI) ; Ri | Richness  Coefficient (95%CI) ; Ri |
| --- | --- | --- | --- | --- |
| Farm related | Building age | 0.91 (0.19 ; 1.64) ; 0.84 |  |  |
|  | Farm area |  |  |  |
|  | Number of pigs | 2.8 (0.58 ; 5.02) ; 0.71 | 2.8 (1.49 ; 4.12) ; 0.9 |  |
|  | Presence of empty rooms | 1.48 (0.36 ; 2.6) ; 0.91 | 1.02 (0.46 ; 1.58) ; 0.98 | 0.41 (0.13 ; 0.7) ; 0.92 |
|  | Presence of illumination | 0.75 (-0.06 ; 1.55) ; 0.64 |  | 0.26 (-0.02 ; 0.55) ; 0.35 |
|  | Presence of irrigation canal |  |  |  |
|  | Presence of space behind gutters |  |  |  |
|  | Presence of shutters |  |  |  |
|  | Presence of hollow trees |  |  |  |
|  | Size of sewage tank |  |  |  |
| Landscape related | Agriculture (proportion) | -6.25 (-15.56 ; 3.06) ; 0.07 | -2.06 (-12.06 ; 7.95) ; 0.22 | -0.53 (-6.57 ; 5.51) ; 0.16 |
|  | Urban (proportion) | -11.83 (-24.65 ; 0.99) ; 0.07 | -6.49 (-18.94 ; 5.96) ; 0.22 | -3.09 (-10.8 ; 4.63) ; 0.16 |
|  | Wood (proportion) | -6.39 (-16.61 ; 3.84) ; 0.45 | -2.9 (-13.34 ; 7.54) ; 0.22 | -1.26 (-7.74 ; 5.23) ; 0.16 |
|  | Water (proportion) |  |  |  |
|  | Distance to water |  |  | -0.7 (-1.39 ; -0.01) ; 0.45 |
|  | Distance to wood | -1.2 (-2.66 ; 0.25) ; 0.39 |  |  |
|  | Number of patches |  | 1.12 (0 ; 2.24) ; 0.08 |  |
