# Supplementary material for: A multi-disciplinary approach to identify spillover interfaces of bat coronaviruses to pig farms in Italy
Source: PLoS One. 2025 Oct 15;20(10):e0332117. doi: 10.1371/journal.pone.0332117 (PMC12527140; doi:10.1371/journal.pone.0332117)
Supplement: S8 Table — (DOCX) [file pone.0332117.s008.docx]

**Table S8. Summary table of virological analysis.** The table shows the total sample size, the number of positive results and the sampling dates of the three colonies investigated trough active surveillance. Different colours for the date of sampling represents the distinction between the two years of sampling, in blue the 2021 sampling season and in orange 2022 sampling season.

| **Colony ID** | **Date of sampling** | **Total sample size**  **(individual + environmental)** | **Positive samples** | **Percentage of positivity** |
| --- | --- | --- | --- | --- |
| *Colony A* | 21/05/2021 | 6 (3+3) | 0 | 0 |
|  | 26/07/2021 | 24 (0+24) | 0 | 0 |
|  | 06/09/2021 | 30 (3+27) | 5 | 16,7 |
|  | 04/05/2022 | 22 (3+19) | 1 | 4,5 |
|  | 14/06/2022 | 22 (2+20) | 0 | 0 |
|  | 09/09/2022 | 9 (7+2) | 0 | 0 |
| Total |  | 113 (18+95) | 6 | 5,3 |
| *Colony B* | 14/05/2022 | 20 (0+20) | 1 | 5 |
|  | 05/06/2022 | 20 (0+20) | 1 | 5 |
|  | 25/06/2022 | 20 (0+20) | 0 | 0 |
|  | 16/07/2022 | 20 (0+20) | 0 | 0 |
|  | 06/08/2022 | 20 (0+20) | 7 | 35 |
|  | 28/08/2022 | 20 (0+20) | 2 | 10 |
| Total |  | 120 | 11 | 9,2 |
| *Colony C* | 07/05/2022 | 20 (0+20) | 13 | 65 |
|  | 28/05/2022 | 20 (0+20) | 7 | 35 |
|  | 25/06/2022 | 20 (0+20) | 3 | 15 |
|  | 16/07/2022 | 20 (0+20) | 0 | 0 |
|  | 08/08/2022 | 20 (0+20) | 4 | 20 |
|  | 28/08/2022 | 20 (0+20) | 11 | 55 |
| Total |  | 120 | 38 | 31,7 |
| **Overall** |  | 353 | 55 | 15,6 |
