# Supplementary material for: A multi-disciplinary approach to identify spillover interfaces of bat coronaviruses to pig farms in Italy
Source: PLoS One. 2025 Oct 15;20(10):e0332117. doi: 10.1371/journal.pone.0332117 (PMC12527140; doi:10.1371/journal.pone.0332117)
Supplement: S9 Table — (DOCX) [file pone.0332117.s009.docx]

**Table S9. Sequences used for phylogenetic analyses**. Novel sequences from this study are shown in bold.

| **Genbank a.n.** | **isolate name** | **sampling location** | **sampling year** | **host species** |
| --- | --- | --- | --- | --- |
| KT368860 | camel/Jeddah/Jd6(b)/2015 | Saudi_Arabia | 2015 | *Camelus_dromedarius* |
| OK094448 | Camel/Kenya/M23C07/2019 | Kenia | 2019 | *Camelus_dromedarius* |
| KF312399 | Eptesicus/13RS384_26/Italy/2012 | Italy | 2012 | *Eptesicus serotinus* |
| KY009613 | C090/China/2015 | China | 2015 | *Eptesicus serotinus* |
| KY009618 | C078/China/2015 | China | 2015 | *Eptesicus serotinus* |
| KY009619 | C092/China/2015 | China | 2015 | *Eptesicus serotinus* |
| KY009621 | C074/China/2015 | China | 2015 | *Eptesicus serotinus* |
| KY009624 | C073/China/2015 | China | 2015 | *Eptesicus serotinus* |
| KY009626 | C060/China/2015 | China | 2015 | *Eptesicus serotinus* |
| KY009627 | C059/China/2015 | China | 2015 | *Eptesicus serotinus* |
| KY009628 | C058/China/2015 | China | 2015 | *Eptesicus serotinus* |
| KY009631 | C055/China/2015 | China | 2015 | *Eptesicus serotinus* |
| KY009632 | C056/China/2015 | China | 2015 | *Eptesicus serotinus* |
| KY009634 | C091/China/2015 | China | 2015 | *Eptesicus serotinus* |
| KY432468 | 16BF104 | South_Korea | 2016 | *Eptesicus serotinus* |
| MT364019 | BtCoV/Esh/TW/2016-2654 | Taiwan | 2016 | *Eptesicus serotinus* |
| MT387187 | BtCov/Esh/TW/CYCU-E25 | Taiwan | 2019 | *Eptesicus serotinus* |
| MT387188 | BtCov/Esh/TW/CYCU-E27 | Taiwan | 2019 | *Eptesicus serotinus* |
| OK017772 | JX2020D | China | 2020 | *Eptesicus serotinus* |
| OP562939 | RIBSP-KZ/Aktobe-BatCov-21-2021 | Kazakhstan | 2021 | *Eptesicus serotinus* |
| OP562944 | RIBSP-KZ/WKO-BatCov-55-2021 | Kazakhstan | 2021 | *Eptesicus serotinus* |
| OQ175223 | BtEs-BetaCoV/JX2020-Q242 | China | 2020 | *Eptesicus serotinus* |
| OQ175257 | BtEs-AlphaCoV/JX2020-Q237 | China | 2020 | *Eptesicus serotinus* |
| OQ175258 | BtEs-AlphaCoV/JX2020-Q238 | China | 2020 | *Eptesicus serotinus* |
| OQ175259 | BtEs-AlphaCoV/JX2020-Q239 | China | 2020 | *Eptesicus serotinus* |
| OQ175260 | BtEs-AlphaCoV/JX2020-Q240 | China | 2020 | *Eptesicus serotinus* |
| OQ175263 | BtEs-BetaCoV/JX2020-Q243 | China | 2020 | *Eptesicus serotinus* |
| **Genbank a.n.** | **isolate name** | **sampling location** | **sampling year** | **host species** |
| OQ175388 | BtEs-BetaCoV/JX2020-P38 | China | 2020 | *Eptesicus serotinus* |
| OQ175389 | BtEs-BetaCoV/JX2020-P39 | China | 2020 | *Eptesicus serotinus* |
| OQ175390 | BtEs-BetaCoV/JX2020-P40 | China | 2020 | *Eptesicus serotinus* |
| OQ175391 | BtEs-BetaCoV/JX2020-P41 | China | 2020 | *Eptesicus serotinus* |
| OQ175392 | BtEs-BetaCoV/JX2020-P42 | China | 2020 | *Eptesicus serotinus* |
| OQ175393 | BtEs-AlphaCoV/JX2020-P43 | China | 2020 | *Eptesicus serotinus* |
| KT717386 | Eum.gla/242/BRA/2013 | Brazil | 2013 | *Eumops glaucinus* |
| MK462253 | Hu/Riyadh-KSA-18013832/2018 | Saudi_Arabia | 2018 | *Homo_sapiens* |
| KX442564 | YD131305 | China | 2013 | *Hypsugo pulveratus* |
| HQ184059 | H.sav/J/Spain/2007 | Spain | 2007 | *Hypsugo savii* |
| HQ184059 | H.sav/J/Spain/2007 | Spain | 2007 | *Hypsugo savii* |
| HQ184061 | H.sav/L/Spain/2007 | Spain | 2007 | *Hypsugo savii* |
| KF500940 | H.sav/Italy/206645-40/2011 | Italy | 2011 | *Hypsugo savii* |
| MG596802 | Bat-CoV/H.savii/Italy/206645-40/2011 | Italy | 2011 | *Hypsugo savii* |
| OQ627315 | 247433-2 | Italy | 2020 | *Hypsugo savii* |
| OQ627321 | 378052-3 | Italy | 2020 | *Hypsugo savii* |
| OQ627324 | 297348-34-int | Italy | 2021 | *Hypsugo savii* |
| OQ627326 | 378052-11 | Italy | 2020 | *Hypsugo savii* |
| PV420065 | IZSVe20/99216 | Italy | 2020 | *Hypsugo_savii* |
| PV420066 | IZSVe22/92499 | Italy | 2022 | *Hypsugo_savii* |
| PV420123 | IZSVe18/140928 | Italy | 2018 | *Hypsugo_savii* |
| MG021452 | NL140422 | China | 2014 | *La_io* |
| MG987421 | NL140455 | China | 2014 | *La_io* |
| OQ175267 | BtIl-BetaCoV/YN2020-Q247 | China | 2020 | *La_io* |
| OQ175423 | BtIi-BetaCoV/YN2020-P73 | China | 2020 | *La_io* |
| OQ297692 | GZ/L165.18/2022 | China | 2022 | *La_io* |
| OQ297729 | GZ/L172.18/2022 | China | 2022 | *Myotis chinensis* |
| KY009614 | C081/China/2015 | China | 2015 | *Myotis pequinius* |
| KY009615 | C082/China/2015 | China | 2015 | *Myotis pequinius* |
| KY009622 | C083/China/2015 | China | 2015 | *Myotis pequinius* |
| **Genbank a.n.** | **isolate name** | **sampling location** | **sampling year** | **host species** |
| OQ175139 | BtMr-AlphaCoV/GD2017-Q119 | China | 2017 | *Myotis ricketti* |
| OQ175144 | BtMr-AlphaCoV/GD2017-Q124 | China | 2017 | *Myotis ricketti* |
| OQ175146 | BtMr-AlphaCoV/GD2017-Q126 | China | 2017 | *Myotis ricketti* |
| OQ175147 | BtMr-AlphaCoV/GD2017-Q127 | China | 2017 | *Myotis ricketti* |
| OQ175148 | BtMr-AlphaCoV/GD2017-Q128 | China | 2017 | *Myotis ricketti* |
| OQ175149 | BtMr-AlphaCoV/GD2017-Q129 | China | 2017 | *Myotis ricketti* |
| OQ175150 | BtMr-AlphaCoV/GD2017-Q130 | China | 2017 | *Myotis ricketti* |
| OQ175152 | BtMr-AlphaCoV/GX2017-Q132 | China | 2017 | *Myotis ricketti* |
| OQ175155 | BtMr-AlphaCoV/YN2016-Q135 | China | 2016 | *Myotis ricketti* |
| PQ577594 | HD13625 | China | 2013 | *Myotis ricketti* |
| OQ175131 | BtMa-AlphaCoV/JX2020-Q111 | China | 2020 | *Myotis_adversus* |
| KF312400 | Myotis/13rs384_31/Italy/2012 | Italy | 2012 | *Myotis_blythii* |
| OQ909823 | Bat-Cov/KZ-N3/Myotis_blythii/19-07-2022 | Kazakhstan | 2022 | *Myotis_blythii* |
| OQ909824 | Bat-Cov/KZ-N40/Myotis_blythii/26-07-2022 | Kazakhstan | 2022 | *Myotis_blythii* |
| MN065811 | BtCoV/008_16/M.bra/FIN/2016 | Finland | 2016 | *Myotis_brandtii* |
| OQ175132 | BtMc-AlphaCoV/YN2020-Q112 | China | 2020 | *Myotis_chinensis* |
| GU190216 | NM98-62/GER/2008 | Germany | 2008 | *Myotis_daubentonii* |
| KF569976 | BCMda55 | China | 2011 | *Myotis_daubentonii* |
| KU182956 | JPDB163 | China | 2012 | *Myotis_daubentonii* |
| KU182957 | JPDB168 | China | 2012 | *Myotis_daubentonii* |
| KU182965 | JPDB144 | China | 2012 | *Myotis_daubentonii* |
| KU182973 | JPDB166 | China | 2012 | *Myotis_daubentonii* |
| MG923571 | BtCoV/606_14/M.dau/FIN/2014 | Finland | 2014 | *Myotis_daubentonii* |
| MG923573 | BtCoV/012_16/M.dau/FIN/2016 | Finland | 2016 | *Myotis_daubentonii* |
| MG923574 | BtCoV/020_16/M.dau/FIN/2016 | Finland | 2016 | *Myotis_daubentonii* |
| MN482243 | BtCoV/21164-6/M.dau/DK/2015 | Denmark | 2015 | *Myotis_daubentonii* |
| MN482243 | BtCoV/21164-6/M.dau/DK/2015 | Denmark | 2015 | *Myotis_daubentonii* |
| MN535731 | BtCoV/13585-35/M.dau/DK/2014 | Denmark | 2014 | *Myotis_daubentonii* |
| MN535731 | BtCoV/13585-35/M.dau/DK/2014 | Denmark | 2014 | *Myotis_daubentonii* |
| **Genbank a.n.** | **isolate name** | **sampling location** | **sampling year** | **host species** |
| MN535732 | BtCoV/13585-58/M.dau/DK/2014 | Denmark | 2014 | *Myotis_daubentonii* |
| MN535732 | BtCoV/13585-58/M.dau/DK/2014 | Denmark | 2014 | *Myotis_daubentonii* |
| MN535733 | BtCoV/OV-157/M.dau/DK/2018 | Denmark | 2018 | *Myotis_daubentonii* |
| MN535733 | BtCoV/OV-157/M.dau/DK/2018 | Denmark | 2018 | *Myotis_daubentonii* |
| MZ218052 | BtCoV/21164-6-alt/M.dau/DK/2015 | Denmark | 2015 | *Myotis_daubentonii* |
| MZ218052 | BtCoV/21164-6-alt/M.dau/DK/2015 | Denmark | 2015 | *Myotis_daubentonii* |
| NC_076629 | BtCoV/020_16/M.dau/FIN/2016 | Finland | 2016 | *Myotis_daubentonii* |
| NC_076629 | BtCoV/020_16/M.dau/FIN/2016 | Finland | 2016 | *Myotis_daubentonii* |
| OK663601 | BtCoVBatGuano15_SWE2020 | Sweden | 2020 | *Myotis_daubentonii* |
| OK663601 | BtCoVBatGuano15_SWE2020 | Sweden | 2020 | *Myotis_daubentonii* |
| OQ401250 | MdGB01 | UK | 2020 | *Myotis_daubentonii* |
| OQ401250 | MdGB01 | UK | 2020 | *Myotis_daubentonii* |
| OQ401254 | MdGB02 | UK | 2011 | *Myotis_daubentonii* |
| OQ401254 | MdGB02 | UK | 2021 | *Myotis_daubentonii* |
| OQ401255 | MdGB03 | UK | 2021 | *Myotis_daubentonii* |
| OQ401255 | MdGB03 | UK | 2021 | *Myotis_daubentonii* |
| KY502383 | BtCoV/Myotis emarginatus/LUX/LUX15_A_46/2015 | Luxembourg | 2015 | *Myotis_emarginatus* |
| KY502384 | BtCoV/Myotis emarginatus/LUX/LUX15_A_48/2015 | Luxembourg | 2015 | *Myotis_emarginatus* |
| KY502388 | BtCoV/Myotis emarginatus/LUX/LUX15_A_158/2015 | Luxembourg | 2015 | *Myotis_emarginatus* |
| KY502391 | BtCoV/Myotis emarginatus/LUX/LUX15_A_284/2015 | Luxembourg | 2015 | *Myotis_emarginatus* |
| KY502404 | BtCoV/Myotis emarginatus/LUX/LUX16_A_1082/2016 | Luxembourg | 2016 | *Myotis_emarginatus* |
| KY780397 | Mmyo4658_IT_16 | Italy | 2016 | *Myotis_myotis* |
| KY780398 | Mmyo4663_IT_16 | Italy | 2016 | *Myotis_myotis* |
| **Genbank a.n.** | **isolate name** | **sampling location** | **sampling year** | **host species** |
| ON325309 | BatCoV/M.myotis/Switzerland/2019 | Switzerland | 2019 | *Myotis_myotis* |
| ON325309 | BatCoV/M.myotis/Switzerland/2019 | Switzerland | 2019 | *Myotis_myotis* |
| ON325310 | BatCoV/M.myotis/Switzerland/2021 | Switzerland | 2021 | *Myotis_myotis* |
| KY780381 | Mnat560_IT_13 | Italy | 2013 | *Myotis_nattereri* |
| OM030318 | JSB_DaZu | China | 2016 | *Myotis_ricketti* |
| OQ175040 | BtMs-AlphaCoV/GX2016-Q136 | China | 2017 | *Myotis_ricketti* |
| OQ175134 | BtMr-AlphaCoV/GD2017-Q114 | China | 2017 | *Myotis_ricketti* |
| OQ175136 | BtMr-AlphaCoV/GD2019-Q116 | China | 2019 | *Myotis_ricketti* |
| OQ175137 | BtMr-AlphaCoV/GD2017-Q117 | China | 2017 | *Myotis_ricketti* |
| OQ175138 | BtMr-AlphaCoV/GD2017-Q118 | China | 2017 | *Myotis_ricketti* |
| OQ175140 | BtMr-AlphaCoV/GD2019-Q120 | China | 2019 | *Myotis_ricketti* |
| OQ175141 | BtMr-AlphaCoV/GD2019-Q121 | China | 2019 | *Myotis_ricketti* |
| OQ175142 | BtMr-AlphaCoV/GD2019-Q122 | China | 2019 | *Myotis_ricketti* |
| OQ175143 | BtMr-AlphaCoV/YN2020-Q123 | China | 2020 | *Myotis_ricketti* |
| OQ175152 | BtMr-AlphaCoV/GX2017-Q132 | China | 2017 | *Myotis_ricketti* |
| OQ175153 | BtMr-AlphaCoV/JX2020-Q133 | China | 2020 | *Myotis_ricketti* |
| OQ175154 | BtMr-AlphaCoV/JX2020-Q134 | China | 2020 | *Myotis_ricketti* |
| MG205595 | BtCoV/20150816HFP_NC1/Neoromicia/RSA | South Africa | 2006 | *Neoromicia capensis* |
| MF593268 | Neoromicia/5038 | South_Africa | 2015 | *Neoromicia_capensis* |
| MG817484 | BtCoV/20141103SRP_NC3/Neoromicia/RSA | South_Africa | 2014 | *Neoromicia_capensis* |
| GU190239 | BNM98-30/BGR/2008 | Bulgaria | 2008 | *Nyctalus_leisleri* |
| OQ175694 | BtPa-BetaCoV/GD2016-P344 | China | 2016 | *Pipistrellus abramus* |
| OQ175695 | BtPa-BetaCoV/GD2016-P345 | China | 2016 | *Pipistrellus abramus* |
| OQ175696 | BtPa-BetaCoV/GD2016-P346 | China | 2016 | *Pipistrellus abramus* |
| MG252861 | BtCoV/20140122FEK_PSP1/Pipistrellus/RSA | South_Africa | 2014 | *Pipistrellus hesperidus* |
| MG310224 | BtCoV/20140122FEK_PSP4/Pipistrellus/RSA | South_Africa | 2014 | *Pipistrellus hesperidus* |
| **Genbank a.n.** | **isolate name** | **sampling location** | **sampling year** | **host species** |
| MG310230 | BtCoV/20150312_DC34/Pipistrellus/RSA | South_Africa | 2015 | *Pipistrellus hesperidus* |
| OQ175269 | PaGD16 | China | 2020 | *Pipistrellus_abramus* |
| HQ184058 | P.kuh/Iprima/Spain/2007 | Spain | 2007 | *Pipistrellus_kuhlii* |
| KF493887 | Pikuh/CII_KSA_001/Riyadh/Saudi Arabia/2013 | Saudi_Arabia | 2013 | *Pipistrellus_kuhlii* |
| KF500942 | Italy/206645-3/2011 | Italy | 2011 | *Pipistrellus_kuhlii* |
| KF500943 | kuhlii/Italy/206645-27/2011 | Italy | 2011 | *Pipistrellus_kuhlii* |
| KF500944 | Italy/206645-29/2011 | Italy | 2011 | *Pipistrellus_kuhlii* |
| KF500946 | Pipistrellus kuhlii/Italy/206645-53/2011 | Italy | 2011 | *Pipistrellus_kuhlii* |
| KF500947 | Italy/206645-54/2011 | Italy | 2011 | *Pipistrellus_kuhlii* |
| KF500950 | Italy/330375-15/2012 | Italy | 2012 | *Pipistrellus_kuhlii* |
| KY780383 | Pkuh605_IT_14 | Italy | 2014 | *Pipistrellus_kuhlii* |
| KY780401 | Pkuh600_IT_14 | Italy | 2014 | *Pipistrellus_kuhlii* |
| KY780402 | Pkuh621_IT_14 | Italy | 2014 | *Pipistrellus_kuhlii* |
| MG596803 | Bat-CoV/P.khulii/Italy/206645-63/2011 | Italy | 2011 | *Pipistrellus_kuhlii* |
| MH938448 | Bat-CoV/P.kuhlii/Italy/206645-41/2011 | Italy | 2011 | *Pipistrellus_kuhlii* |
| MH938450 | Bat-CoV/P.kuhlii/Italy/206679-3/2010 | Italy | 2010 | *Pipistrellus_kuhlii* |
| MW089336 | 19RS495-11_ColA18 | Italy | 2018 | *Pipistrellus_kuhlii* |
| NC_046964 | Bat-CoV/P.kuhlii/Italy/3398-19/2015 | Italy | 2015 | *Pipistrellus_kuhlii* |
| OQ249692 | IZSVE_123662/2021 | Italy | 2021 | *Pipistrellus_kuhlii* |
| OR052074 | Bat-CoV/RU/ROV21-131/1 | Russia | 2021 | *Pipistrellus_kuhlii* |
| OR052075 | Bat-CoV/RU/ROV21-132/1 | Russia | 2021 | *Pipistrellus_kuhlii* |
| OR052076 | Bat-CoV/RU/ROV21-132/2 | Russia | 2021 | *Pipistrellus_kuhlii* |
| OR147948 | Bat-CoV/RU/ROV21-132/4-Ped | Russia | 2021 | *Pipistrellus_kuhlii* |
| PP976053 | BH35/18_85 | Austria | 2018 | *Pipistrellus_kuhlii* |
| PP976055 | BH35/18_351 | Austria | 2018 | *Pipistrellus_kuhlii* |
| **Genbank a.n.** | **isolate name** | **sampling location** | **sampling year** | **host species** |
| PQ439331 | BtCoV/PK104/Rus/Rostov-on-Don/2023 | Russia | 2023 | *Pipistrellus_kuhlii* |
| PQ439332 | BtCoV/PK116/Rus/Rostov-on-Don/2023 | Russia | 2023 | *Pipistrellus_kuhlii* |
| PQ439333 | BtCoV/PK139/Rus/Rostov-on-Don/2023 | Russia | 2023 | *Pipistrellus_kuhlii* |
| PV420067 | IZSVe21/123662 | Italy | 2021 | *Pipistrellus_kuhlii* |
| PV420068 | IZSVe21/140233-2_ColA921 | Italy | 2021 | *Pipistrellus_kuhlii* |
| PV420069 | IZSVe21/140233-23_ColA921 | Italy | 2021 | *Pipistrellus_kuhlii* |
| PV420070 | IZSVe21/140233-22_ColA921 | Italy | 2021 | *Pipistrellus_kuhlii* |
| PV420071 | IZSVe21/140233-4_ColA921 | Italy | 2021 | *Pipistrellus_kuhlii* |
| PV420072 | IZSVe21/140233-7_ColA921 | Italy | 2021 | *Pipistrellus_kuhlii* |
| PV420073 | IZSVe22/91389-1_ColB522 | Italy | 2022 | *Pipistrellus_kuhlii* |
| PV420074 | IZSVe22/90604-2_ColB622 | Italy | 2022 | *Pipistrellus_kuhlii* |
| PV420075 | IZSVe22/113477-11_ColB8a22 | Italy | 2022 | *Pipistrellus_kuhlii* |
| PV420076 | IZSVe22/113477-13_ColB8a22 | Italy | 2022 | *Pipistrellus_kuhlii* |
| PV420077 | IZSVe22/113477-3_ColB8a22 | Italy | 2022 | *Pipistrellus_kuhlii* |
| PV420078 | IZSVe22/113477-7_ColB8a22 | Italy | 2022 | *Pipistrellus_kuhlii* |
| PV420079 | IZSVe22/113477-15_ColB8a22 | Italy | 2022 | *Pipistrellus_kuhlii* |
| PV420080 | IZSVe22/113477-16_ColB8a22 | Italy | 2022 | *Pipistrellus_kuhlii* |
| PV420081 | IZSVe22/113477-2_ColB8a22 | Italy | 2022 | *Pipistrellus_kuhlii* |
| PV420082 | IZSVe22/113480-9_ColB8b22 | Italy | 2022 | *Pipistrellus_kuhlii* |
| PV420083 | IZSVe22/113480-15_ColB8b22 | Italy | 2022 | *Pipistrellus_kuhlii* |
| PV420084 | IZSVe22/56664-1_ColC422 | Italy | 2022 | *Pipistrellus_kuhlii* |
| PV420085 | IZSVe22/90067-1_ColC5a22 | Italy | 2022 | *Pipistrellus_kuhlii* |
| PV420086 | IZSVe22/90067-13_ColC5a22 | Italy | 2022 | *Pipistrellus_kuhlii* |
| PV420087 | IZSVe22/90067-14_ColC5a22 | Italy | 2022 | *Pipistrellus_kuhlii* |
| PV420088 | IZSVe22/90067-15_ColC5a22 | Italy | 2022 | *Pipistrellus_kuhlii* |
| PV420089 | IZSVe22/90067-16_ColC5a22 | Italy | 2022 | *Pipistrellus_kuhlii* |
| PV420090 | IZSVe22/90067-19_ColC5a22 | Italy | 2022 | *Pipistrellus_kuhlii* |
| PV420091 | IZSVe22/90067-2_ColC5a22 | Italy | 2022 | *Pipistrellus_kuhlii* |
| **Genbank a.n.** | **isolate name** | **sampling location** | **sampling year** | **host species** |
| PV420092 | IZSVe22/90067-3_ColC5a22 | Italy | 2022 | *Pipistrellus_kuhlii* |
| PV420093 | IZSVe22/90067-6_ColC5a22 | Italy | 2022 | *Pipistrellus_kuhlii* |
| PV420094 | IZSVe22/90067-7_ColC5a22 | Italy | 2022 | *Pipistrellus_kuhlii* |
| PV420095 | IZSVe22/90067-20_ColC5a22 | Italy | 2022 | *Pipistrellus_kuhlii* |
| PV420096 | IZSVe22/90067-4_ColC5a22 | Italy | 2022 | *Pipistrellus_kuhlii* |
| PV420097 | IZSVe22/90067-12_ColC5a22 | Italy | 2022 | *Pipistrellus_kuhlii* |
| PV420098 | IZSVe22/92366-1_ColC5b22 | Italy | 2022 | *Pipistrellus_kuhlii* |
| PV420099 | IZSVe22/92366-10_ColC5b22 | Italy | 2022 | *Pipistrellus_kuhlii* |
| PV420100 | IZSVe22/92366-12_ColC5b22 | Italy | 2022 | *Pipistrellus_kuhlii* |
| PV420101 | IZSVe22/92366-15_ColC5b22 | Italy | 2022 | *Pipistrellus_kuhlii* |
| PV420102 | IZSVe22/92366-4_ColC5b22 | Italy | 2022 | *Pipistrellus_kuhlii* |
| PV420103 | IZSVe22/92366-7_ColC5b22 | Italy | 2022 | *Pipistrellus_kuhlii* |
| PV420104 | IZSVe22/92366-8_ColC5b22 | Italy | 2022 | *Pipistrellus_kuhlii* |
| PV420105 | IZSVe22/112985-1_ColC622 | Italy | 2022 | *Pipistrellus_kuhlii* |
| PV420106 | IZSVe22/112985-7_ColC622 | Italy | 2022 | *Pipistrellus_kuhlii* |
| PV420107 | IZSVe22/112985-15_ColC622 | Italy | 2022 | *Pipistrellus_kuhlii* |
| PV420108 | IZSVe22/113478-6_ColC8a22 | Italy | 2022 | *Pipistrellus_kuhlii* |
| PV420109 | IZSVe22/113478-8_ColC8a22 | Italy | 2022 | *Pipistrellus_kuhlii* |
| PV420110 | IZSVe22/113478-9_ColC8a22 | Italy | 2022 | *Pipistrellus_kuhlii* |
| PV420111 | IZSVe22/113478-19_ColC8a22 | Italy | 2022 | *Pipistrellus_kuhlii* |
| PV420112 | IZSVe22/113482-16_ColC8b22 | Italy | 2022 | *Pipistrellus_kuhlii* |
| PV420113 | IZSVe22/113482-1_ColC8b22 | Italy | 2022 | *Pipistrellus_kuhlii* |
| PV420114 | IZSVe22/113482-10_ColC8b22 | Italy | 2022 | *Pipistrellus_kuhlii* |
| PV420115 | IZSVe22/113482-12_ColC8b22 | Italy | 2022 | *Pipistrellus_kuhlii* |
| PV420116 | IZSVe22/113482-4_ColC8b22 | Italy | 2022 | *Pipistrellus_kuhlii* |
| PV420117 | IZSVe22/113482-5_ColC8b22 | Italy | 2022 | *Pipistrellus_kuhlii* |
| PV420118 | IZSVe22/113482-6_ColC8b22 | Italy | 2022 | *Pipistrellus_kuhlii* |
| PV420119 | IZSVe22/113482-8_ColC8b22 | Italy | 2022 | *Pipistrellus_kuhlii* |
| PV420120 | IZSVe22/113482-19_ColC8b22 | Italy | 2022 | *Pipistrellus_kuhlii* |
| PV420121 | IZSVe22/113482-17_ColC8b22 | Italy | 2022 | *Pipistrellus_kuhlii* |
| **Genbank a.n.** | **isolate name** | **sampling location** | **sampling year** | **host species** |
| PV420122 | IZSVe22/113482-20_ColC8b22 | Italy | 2022 | *Pipistrellus_kuhlii* |
| OP919651 | MOW15-21/2015 | Russia | 2015 | *Pipistrellus_nathusii* |
| OQ230639 | MOW15-23/2015 | Russia | 2015 | *Pipistrellus_nathusii* |
| OQ348397 | BtCoV/P.nathusii/NL/2018-34 | Netherlands | 2018 | *Pipistrellus_nathusii* |
| GQ259964 | P.pipi/VM312/2008/NLD | Netherlands | 2008 | *Pipistrellus_pipistrellus* |
| GQ259977 | P.pipi/VM314/2008/NLD | Netherlands | 2008 | *Pipistrellus_pipistrellus* |
| KT345294 | Pip1_Cr_FR_2014 | France | 2014 | *Pipistrellus_pipistrellus* |
| KT345295 | Pip2_Cr_FR_2014 | France | 2014 | *Pipistrellus_pipistrellus* |
| KT345296 | Pip3_M_FR_2014 | France | 2014 | *Pipistrellus_pipistrellus* |
| KX285197 | PREDICT-EHA-156-12-NL13847 | China | 2013 | *Pipistrellus_pipistrellus* |
| KX285198 | PREDICT_CoV-60/EHA-156-12-NL13847 | China | 2013 | *Pipistrellus_pipistrellus* |
| KX285199 | PREDICT-EHA-156-12-NL13849 | China | 2013 | *Pipistrellus_pipistrellus* |
| KX285207 | PREDICT_CoV-57/EHA-156-12-NL13884 | China | 2013 | *Pipistrellus_pipistrellus* |
| KX285208 | PREDICT_CoV-57/EHA-156-12-NL13892 | China | 2013 | *Pipistrellus_pipistrellus* |
| KX285209 | PREDICT_CoV-57/EHA-156-12-NL13893 | China | 2013 | *Pipistrellus_pipistrellus* |
| KX285210 | PREDICT_CoV-61/EHA-156-12-NL13893 | China | 2013 | *Pipistrellus_pipistrellus* |
| KX285211 | PREDICT_CoV-61/EHA-156-12-NL13897 | China | 2013 | *Pipistrellus_pipistrellus* |
| KX285212 | PREDICT_CoV-61/EHA-156-12-NL13901 | China | 2013 | *Pipistrellus_pipistrellus* |
| KX285213 | PREDICT_CoV-61/EHA-156-12-NL13903 | China | 2013 | *Pipistrellus_pipistrellus* |
| KY780384 | Ppip1000_IT_14 | Italy | 2014 | *Pipistrellus_pipistrellus* |
| **Genbank a.n.** | **isolate name** | **sampling location** | **sampling year** | **host species** |
| KY780385 | Ppip1015C_IT_14 | Italy | 2014 | *Pipistrellus_pipistrellus* |
| KY780386 | Ppip1016_IT_14 | Italy | 2014 | *Pipistrellus_pipistrellus* |
| MH921428 | CCPaC51 | China | 2016 | *Pipistrellus_pipistrellus* |
| MH921429 | CCPaC52 | China | 2016 | *Pipistrellus_pipistrellus* |
| OQ134956 | 77843_2021 | Italy | 2021 | *Pipistrellus_pipistrellus* |
| OQ134957 | 77850_2021 | Italy | 2021 | *Pipistrellus_pipistrellus* |
| OQ134958 | 60649_2020_5114 | Italy | 2020 | *Pipistrellus_pipistrellus* |
| OQ134959 | 60649_2020_7243 | Italy | 2020 | *Pipistrellus_pipistrellus* |
| OQ401253 | PpiGB01 | UK | 2005 | *Pipistrellus_pipistrellus* |
| OR625571 | F1_2023 | Portugal | 2023 | *Pipistrellus_pipistrellus* |
| ON457561 | BtCoV/F-MV2/P.pyg/SWE/2020 | Sweden | 2020 | *Pipistrellus_pygmaeus* |
| KY780396 | Paur4241_IT_16 | Italy | 2016 | *Plecotus auritus* |
| OQ401251 | PaGB01 | UK | 2020 | *Plecotus_auritus* |
| DQ412042 | Rf1 | China | 2004 | *Rhinolophus ferrumequinum* |
| KC633199 | BtCoV/Rhi_fer/It17/ITA/2009 | Italy | 2009 | *R. ferrumequinum* |
| KC633200 | BtCoV/Rhi_fer/It2/ITA/2009 | Italy | 2009 | *R. ferrumequinum* |
| KC633205 | BtCoV/Rhi_fer/It13/ITA/2009 | Italy | 2009 | *R. ferrumequinum* |
| KC633206 | BtCoV/Rhi_fer/It15/ITA/2009 | Italy | 2009 | *R. ferrumequinum* |
| KC633207 | BtCoV/Rhi_fer/FR0711-B3/FRA/2011 | France | 2011 | *R. ferrumequinum* |
| KC633208 | BtCoV/Rhi_fer/FR0711-B11/FRA/2011 | France | 2011 | *R. ferrumequinum* |
| KJ473811 | BtRf-BetaCoV/JL2012 | China | 2012 | *R. ferrumequinum* |
| KJ473812 | BtRf-BetaCoV/HeB2013 | China | 2013 | *R. ferrumequinum* |
| **Genbank a.n.** | **isolate name** | **sampling location** | **sampling year** | **host species** |
| KJ473813 | BtRf-BetaCoV/SX2013 | China | 2013 | *R. ferrumequinum* |
| KP886808 | YNLF_31C | China | 2013 | *R. ferrumequinum* |
| KP886809 | YNLF_34C | China | 2013 | *R. ferrumequinum* |
| KU182964 | JTMC15 | China | 2013 | *R. ferrumequinum* |
| KY770860 | Jiyuan-84 | China | 2012 | *R. ferrumequinum* |
| KY780388 | Rfer4009_IT_16 | Italy | 2016 | *R. ferrumequinum* |
| KY780389 | Rfer4011_IT_16 | Italy | 2016 | *R. ferrumequinum* |
| KY780392 | Rfer4024_IT_16 | Italy | 2016 | *R. ferrumequinum* |
| KY780394 | Rfer4027_IT_16 | Italy | 2016 | *R. ferrumequinum* |
| KY780400 | Rfer4675_IT_2016 | Italy | 2016 | *R. ferrumequinum* |
| KY938558 | 16BO133 | south korea | 2016 | *R. ferrumequinum* |
| LC810614 | YB8_R | Japan | 2021 | *R. ferrumequinum* |
| LC810619 | YB44_R | Japan | 2021 | *R. ferrumequinum* |
| LC810621 | YB9_O | Japan | 2021 | *R. ferrumequinum* |
| MK991904 | B16-75 | South_Korea | 2016 | *R. ferrumequinum* |
| MK991925 | B17-155 | South Korea | 2017 | *R. ferrumequinum* |
| MK991928 | B18-38 | South_Korea | 2018 | *R. ferrumequinum* |
| MK991930 | B18-50 | South Korea | 2018 | *R. ferrumequinum* |
| MK991940 | B18-118 | South_Korea | 2018 | *R. ferrumequinum* |
| MK991942 | B18-148 | South_Korea | 2018 | *R. ferrumequinum* |
| MK991943 | B18-149 | South_Korea | 2018 | *R. ferrumequinum* |
| MK991949 | B18-184 | South_Korea | 2018 | *R. ferrumequinum* |
| MN823618 | Algeria/2017 | Algeria | 2017 | *R. ferrumequinum* |
| MT063921 | PREDICT_CoV-65 | Jordann | 2018 | *R. ferrumequinum* |
| MW880969 | Beta-CoV/bat/LBN/LB20-CO-BAT-37A/2020 | Lebanon | 2020 | *R. ferrumequinum* |
| MZ190137 | BtCoV/Khosta-1/Rh/Russia/2020 | Russia | 2020 | *R. ferrumequinum* |
| MZ893218 | Swarming2018_FT18 | France | 2018 | *R. ferrumequinum* |
| NC_028814 | BtRf-AlphaCoV/HuB2013 | China | 2013 | *R. ferrumequinum* |
| NC_028824 | BtRf-AlphaCoV/YN2012 | China | 2012 | *R. ferrumequinum* |
| OK017758 | ZJ2021B | China | 2021 | *R. ferrumequinum* |
| **Genbank a.n.** | **isolate name** | **sampling location** | **sampling year** | **host species** |
| OK017794 | LN2020A | China | 2020 | *R. ferrumequinum* |
| OK017795 | LN2020B | China | 2020 | *R. ferrumequinum* |
| OK017796 | LN2020C | China | 2020 | *R. ferrumequinum* |
| OK017797 | LN2020E | China | 2020 | *R. ferrumequinum* |
| OK017798 | LN2020F | China | 2020 | *R. ferrumequinum* |
| OK017799 | LN2020G | China | 2020 | *R. ferrumequinum* |
| OK017800 | LN2020H | China | 2020 | *R. ferrumequinum* |
| OL791325 | GE_CoV58_Rhi_fer | Georgia | 2014 | *R. ferrumequinum* |
| ON378802 | BatCoV_B20-50 | South Korea | 2020 | *R. ferrumequinum* |
| ON378803 | BatCoV_B20-97 | South Korea | 2020 | *R. ferrumequinum* |
| ON378807 | BatCoV_B20-180 | South Korea | 2021 | *R. ferrumequinum* |
| OQ175057 | BtRf-AlphaCoV/YN2020-Q37 | China | 2020 | *R. ferrumequinum* |
| OQ175058 | BtRf-AlphaCoV/HB2020-Q38 | China | 2020 | *R. ferrumequinum* |
| OQ175059 | BtRf-AlphaCoV/ZJ2020-Q39 | China | 2020 | *R. ferrumequinum* |
| OQ175060 | BtRf-AlphaCoV/LN2020-Q40 | China | 2020 | *R. ferrumequinum* |
| OQ175061 | BtRf-AlphaCoV/LN2020-Q41 | China | 2020 | *R. ferrumequinum* |
| OQ175062 | BtRf-AlphaCoV/LN2020-Q42 | China | 2020 | *R. ferrumequinum* |
| OQ175063 | BtRf-AlphaCoV/ZJ2020-Q43 | China | 2020 | *R. ferrumequinum* |
| OQ175064 | BtRf-AlphaCoV/ZJ2020-Q44 | China | 2020 | *R. ferrumequinum* |
| OQ175215 | BtRf-AlphaCoV/ZJ2020-Q23 | China | 2020 | *R. ferrumequinum* |
| OQ175222 | BtRf-AlphaCoV/ZJ2020-Q24 | China | 2020 | *R. ferrumequinum* |
| OQ401247 | RfGB01 | UK | 2021 | *R. ferrumequinum* |
| OQ401249 | RfGB02 | UK | 2021 | *R. ferrumequinum* |
| OQ613363 | F30 | Portugal | 2022 | *R. ferrumequinum* |
| PP265313 | BtCoV/Rhi_fer-6/RUS/2022 | Russia | 2022 | *R. ferrumequinum* |
| PP265316 | BtCoV/Rhi_fer-3/RUS/2023 | Russia | 2023 | *R. ferrumequinum* |
| PQ649435 | BtCoV/Rf/Russia/F3/2020 | Russia | 2020 | *R. ferrumequinum* |
| KC633209 | BtCoV/Rhi_hip/R8-09/SPA/2010 | Spain | 2010 | *Rhinolophus hipposideros* |
| KC633220 | BtCoV/Rhi_hip/Slo52/SLO/2009 | Slovenia | 2009 | *R. hipposideros* |
| KF500952 | hipposideros/Italy/187632-2/2012 | Italy | 2012 | *R. hipposideros* |
| **Genbank a.n.** | **isolate name** | **sampling location** | **sampling year** | **host species** |
| KF500953 | hipposideros/Italy/196814/2011 | Italy | 2011 | *R. hipposideros* |
| KF500954 | hipposideros/Italy/243585/2012 | Italy | 2012 | *R. hipposideros* |
| KJ652330 | BtCoV/M9/HUN/2013 | Hungary | 2013 | *R. hipposideros* |
| MW719567 | RhGB01 | UK | 2020 | *R. hipposideros* |
| MZ190138 | BtCoV/Khosta-2/Rh/Russia/2020 | Russia | 2020 | *R. hipposideros* |
| OP776338 | RhGB02 | UK | 2021 | *R. hipposideros* |
| OP776339 | RhGB05 | UK | 2021 | *R. hipposideros* |
| OP776340 | RhGB06 | UK | 2021 | *R. hipposideros* |
| OQ401248 | RhGB07_2-30B | UK | 2020 | *R. hipposideros* |
| MN611522 | SADS_like_HKU2/160660 | China | 2018 | *rhinolophus_affinis* |
| EF203065 | SADS_like_HKU2/HK/46/2006 | China | 2006 | *Rhinolophus_sp* |
| EF203066 | SADS_like_HKU2/HK/298/2006 | China | 2006 | *Rhinolophus_sp* |
| EF203067 | SADS_like_HKU2/HK/33/2006 | China | 2006 | *Rhinolophus_sp* |
| MF370205 | SADS_like_HKU2/GD-01/2017/P2 | China | 2017 | *Rhinolophus_sp* |
| NC_009988 | SADS_like_HKU2/GD/430/2006 | China | 2006 | *Rhinolophus_sp* |
| PP681138 | VS-G11 | Taiwan | 2021 | *Vespertilio sinensis* |
| ON325307 | BatCoV/V.murinus/Switzerland/2020 | Switzerland | 2020 | *Vespertilio_murinus* |
| LC706863 | VsCoV-kj15 | Japan | 2021 | *Vespertilio_sinensis* |
| KJ473821 | BtVs-BetaCoV/SC2013 | China | 2013 | *Vespertilio_superans* |
| MW685622 | PDCoV/Haiti/Human/0081-4/2014 | Hawaii | 2014 | *Homo_sapiens* |
| MW685623 | PDCoV/Haiti/Human/0256-1/2015 | Hawaii | 2015 | *Homo_sapiens* |
| MW685624 | PDCoV/Haiti/Human/0329-4/2015 | Hawaii | 2015 | *Homo_sapiens* |
| DQ011855 | PHEV_VW572 | Belgium | nd | *Sus_scrofa* |
| FJ755618 | TGEV_H16 | China | 1973 | *Sus_scrofa* |
| KC962433 | TGEV_TGEV-HX | China | 2012 | *Sus_scrofa* |
| KJ567050 | PDCoV/8734/USA-IA/2014 | USA | 2014 | *Sus_scrofa* |
| KJ584359 | PDCoV_NE3579 | USA | 2014 | *Sus_scrofa* |
| KM820765 | PDCoV_KNU14-04 | South Korea | 2014 | *Sus_scrofa* |
| KP202848 | TGEV_SHXB | China | 2013 | *Sus_scrofa* |
| **Genbank a.n.** | **isolate name** | **sampling location** | **sampling year** | **host species** |
| KP403954 | PEDV_Ukraine/Poltava01/2014 | Ukraine | 2014 | *Sus_scrofa* |
| KP688354 | PEDV_Hawaii/39249/2014 | Hawaii | 2014 | *Sus_scrofa* |
| KP757890 | PDCoV_CHN-AH-2004 | China | 2004 | *Sus_scrofa* |
| KR003452 | PEDV_15V010/BEL/2015 | Belgium | 2015 | *Sus_scrofa* |
| KR011756 | PEDV_FR/001/2014 | France | 2014 | *Sus_scrofa* |
| KR061458 | PEDV_Italy/7239/2009 | Italy | 2009 | *Sus_scrofa* |
| KR061459 | SeCoV_Italy/213306/2009 | Italy | 2009 | *Sus_scrofa* |
| KR150443 | PDCoV_USA/Arkansas61/2015 | USA | 2015 | *Sus_scrofa* |
| KR265831 | PEDV_CAN/Quebec334/2014 | Canada | 2014 | *Sus_scrofa* |
| KR270796 | PRCV_OH7269 | USA | 2014 | *Sus_scrofa* |
| KT266822 | PDCoV_CH/Sichuan/S27/2012 | China | 2012 | *Sus_scrofa* |
| KU051641 | PDCoV_Swine/Thailand/S5011/2015 | Thailand | 2015 | *Sus_scrofa* |
| KU051649 | PDCoV_Swine/Thailand/S5015L/2015 | Thailand | 2015 | *Sus_scrofa* |
| KU297956 | PEDV_SLO/JH-11/2015 | Slovenia | 2015 | *Sus_scrofa* |
| KU665558 | PDCoV_CHN-LYG-2014 | China | 2014 | *Sus_scrofa* |
| KU729220 | TGEV_TH-98 | China | 1998 | *Sus_scrofa* |
| KU893862 | PEDV_PC22A-P20 | USA | 2013 | *Sus_scrofa* |
| KU893864 | PEDV_PC22A-P40 | USA | 2014 | *Sus_scrofa* |
| KU984334 | PDCoV_TT_1115 | Thailand | 2015 | *Sus_scrofa* |
| KX022602 | PDCoV_USA/Iowa136/2015 | USA | 2015 | *Sus_scrofa* |
| KX022603 | PDCoV_USA/Minnesota140/2015 | USA | 2015 | *Sus_scrofa* |
| KX083668 | TGEV_HE-1 | China | 2015 | *Sus_scrofa* |
| KX118627 | PDCoV_P1_16_BTL_0115/PDCoV/2016/Lao | Laos | 2016 | *Sus_scrofa* |
| KX289955 | PEDV_HUN/5031/2016 | Hungary | 2016 | *Sus_scrofa* |
| KX499468 | TGEV_P115 | China | 2015 | *Sus_scrofa* |
| KX834351 | PDCoV_Swine/Vietnam/HaNoi6/2015 | Vietnam | 2015 | *Sus_scrofa* |
| KX834352 | PDCoV_Swine/Vietnam/Binh21/2015 | Vietnam | 2015 | *Sus_scrofa* |
| **Genbank a.n.** | **isolate name** | **sampling location** | **sampling year** | **host species** |
| KX900393 | TGEV_USA/Z/1986 | USA | 2006 | *Sus_scrofa* |
| KX900394 | TGEV_USA/HB/1988 | USA | 1988 | *Sus_scrofa* |
| KX900395 | TGEV_USA/Minnesota138/2006 | USA | 2006 | *Sus_scrofa* |
| KX900396 | TGEV_USA/Illinois139/2006 | USA | 2006 | *Sus_scrofa* |
| KX900397 | TGEV_USA/NorthCarolina140/2007 | USA | 2007 | *Sus_scrofa* |
| KX900398 | TGEV_USA/Minnesota141/2007 | USA | 2007 | *Sus_scrofa* |
| KX900399 | TGEV_USA/NorthCarolina142/2007 | USA | 2007 | *Sus_scrofa* |
| KX900400 | TGEV_USA/Iowa143/2008 | USA | 2008 | *Sus_scrofa* |
| KX900401 | TGEV_USA/Tennessee144/2008 | USA | 2008 | *Sus_scrofa* |
| KX900402 | TGEV_Mex/145/2008 | USA | 2008 | *Sus_scrofa* |
| KX900403 | TGEV_USA/Illinois146/2008 | USA | 2008 | *Sus_scrofa* |
| KX900404 | TGEV_USA/Oklahoma147/2012 | USA | 2012 | *Sus_scrofa* |
| KX900405 | TGEV_USA/Minnesota148/2013 | USA | 2013 | *Sus_scrofa* |
| KX900406 | TGEV_USA/Illinois149/2013 | USA | 2013 | *Sus_scrofa* |
| KX900407 | TGEV_USA/Minnesota150/2013 | USA | 2013 | *Sus_scrofa* |
| KX900408 | TGEV_USA/Wisconsin151/2014 | USA | 2014 | *Sus_scrofa* |
| KX900409 | TGEV_USA/Minnesota152/2014 | USA | 2014 | *Sus_scrofa* |
| KX900410 | TGEV_USA/Minnesota153/2014 | USA | 2014 | *Sus_scrofa* |
| KX900411 | TGEV_USA/SouthDakota154/2014 | USA | 2014 | *Sus_scrofa* |
| KX998969 | PDCoV_P29_15_VN_1215 | Vietnam | 2015 | *Sus_scrofa* |
| KY019623 | PEDV_SLOreBAS-1/2015 | Slovenia | 2015 | *Sus_scrofa* |
| KY019624 | PEDV_SLOreBAS-2/2015 | Slovenia | 2015 | *Sus_scrofa* |
| KY111278 | PEDV_1842/2016 ITA | Italy | 2016 | *Sus_scrofa* |
| KY354363 | PDCoV_DH1 | South Korea | 2016 | *Sus_scrofa* |
| KY354364 | PDCoV_DH2 | South Korea | 2016 | *Sus_scrofa* |
| KY406735 | PRCV_USA/Minnesota-46140/16 | USA | 2016 | *Sus_scrofa* |
| KY419103 | PHEV_15TOSU25049/2015 | USA | 2015 | *Sus_scrofa* |
| KY419104 | PHEV_15TOSU0331/2015 | USA | 2015 | *Sus_scrofa* |
| **Genbank a.n.** | **isolate name** | **sampling location** | **sampling year** | **host species** |
| KY419105 | PHEV_15TOSU0582/2015 | USA | 2015 | *Sus_scrofa* |
| KY419106 | PHEV/pig/USA/15TOSU1785/2015 | USA | 2015 | *Sus_scrofa* |
| KY419107 | PHEV/pig/USA/15TOSU1209/2015 | USA | 2015 | *Sus_scrofa* |
| KY419109 | PHEV_pig/USA/15TOSU1655/2015 | USA | 2015 | *Sus_scrofa* |
| KY419110 | PHEV_15TOSU1582/2015 | USA | 2015 | *Sus_scrofa* |
| KY419111 | PHEV /pig/USA/15TOSU1727/15 | USA | 2015 | *Sus_scrofa* |
| KY419112 | PHEV_15TOSU1765/2015 | USA | 2015 | *Sus_scrofa* |
| KY419113 | PHEV_USA-15TOSU1582 | USA | 2015 | *Sus_scrofa* |
| KY513724 | PDCoV_CH/Hunan/2014 | China | 2014 | *Sus_scrofa* |
| KY513725 | PDCoV_CH/Jiangsu/2014 | China | 2014 | *Sus_scrofa* |
| KY994645 | PHEV_JL/2008 | China | 2008 | *Sus_scrofa* |
| LC063810 | PEDV_AOM-1/JPN/2014 | Japan | 2014 | *Sus_scrofa* |
| LC063811 | PEDV_FKO-1/JPN/2014 | Japan | 2014 | *Sus_scrofa* |
| LC063812 | PEDV_EHM-1/JPN/2014 | Japan | 2014 | *Sus_scrofa* |
| LC260038 | PDCoV_AKT/JPN/2014 | Japan | 2014 | *Sus_scrofa* |
| LC260039 | PDCoV_GNM-1/JPN/2014 | Japan | 2014 | *Sus_scrofa* |
| LC260040 | PDCoV_GNM-2/JPN/2014 | Japan | 2014 | *Sus_scrofa* |
| LR812926 | PEDV_GER/L03209/2019 | Germany | 2019 | *Sus_scrofa* |
| LR812927 | PEDV_GER/L03206/2019 | Germany | 2019 | *Sus_scrofa* |
| LR812932 | PEDV_GER/L03205/2019 | Germany | 2019 | *Sus_scrofa* |
| LT545990 | SeCoV-GER_L00930_2012 | Germany | 2012 | *Sus_scrofa* |
| LT898418 | PEDV_AUSTRIA_L01065-M10_15-04_2015 | Austria | 2015 | *Sus_scrofa* |
| LT898433 | PEDV_AUSTRIA_L01063-M10_15-02_2015 | Austria | 2015 | *Sus_scrofa* |
| LT898435 | PEDV_ROMANIA_L01330-K25_15-02_2015 | Romania | 2015 | *Sus_scrofa* |
| LT898436 | PEDV_ROMANIA_L01329-K25_15-01_2015 | Romania | 2015 | *Sus_scrofa* |
| **Genbank a.n.** | **isolate name** | **sampling location** | **sampling year** | **host species** |
| LT898441 | PEDV_AUSTRIA_L01064-M10_15-03_2015 | Austria | 2015 | *Sus_scrofa* |
| MF083115 | PHEV_CC14 | China | 2014 | *Sus_scrofa* |
| MF094681 | SADS_isolate_FarmA | China | 2016 | *Sus_scrofa* |
| MF094682 | SADS_isolate_FarmB | China | 2016 | *Sus_scrofa* |
| MF094683 | SADS_isolate_FarmC | China | 2016 | *Sus_scrofa* |
| MF431742 | PDCoV_GD | China | 2015 | *Sus_scrofa* |
| MF577027 | PEDV_Belgorod/dom/2008 | Russia | 2008 | *Sus_scrofa* |
| MF769416 | SADS_isolate 57 | China | 2017 | *Sus_scrofa* |
| MF769417 | SADS_isolate 31 | China | 2017 | *Sus_scrofa* |
| MF769419 | SADS_isolate 197 | China | 2017 | *Sus_scrofa* |
| MH004412 | PEDV_MEX/GTO/02/2016 | Mexico | 2016 | *Sus_scrofa* |
| MH615810 | SADS_CH-FJWT-2018 | China | 2018 | *Sus_scrofa* |
| MK071619 | PEDV_COL/Antioquia00265/2015 | Colombia | 2015 | *Sus_scrofa* |
| MK071621 | PEDV_COL/Cundinamarca3267/14 | Colombia | 2014 | *Sus_scrofa* |
| MK071622 | PEDV_COL/Huila/2014 | Colombia | 2014 | *Sus_scrofa* |
| MK355396 | PDCoV_CHN-SC2015 | China | 2015 | *Sus_scrofa* |
| MK651076 | SADS_GDLX/2019 | China | 2019 | *Sus_scrofa* |
| MK993519 | PDCoV_CHN/Sichuan/2019 | China | 2019 | *Sus_scrofa* |
| MN056942 | PEDV_FR2019001 | France | 2019 | *Sus_scrofa* |
| MN692770 | SeCoV-1480-Murcia-Lorca | Spain | 2014 | *Sus_scrofa* |
| MN692771 | PEDV_1453-Zaragoza-Tauste | Spain | 2014 | *Sus_scrofa* |
| MN692774 | PEDV_481-Pamplona-Tudela | Spain | 2014 | *Sus_scrofa* |
| MN942260 | PDCoV_HeN/swine/2015 | China | 2015 | *Sus_scrofa* |
| MT294722 | SADS_JX-CH/2018 | China | 2018 | *Sus_scrofa* |
| MT490315 | PEDV_EdoMex/103/2018 | Mexico | 2018 | *Sus_scrofa* |
| MT490316 | PEDV_EdoMex/205/2018 | Mexico | 2018 | *Sus_scrofa* |
| MT576083 | TGEV_HQ2016 | China | 2016 | *Sus_scrofa* |
| MT602520 | PEDV_Pig-wt/ESP/Calaf-1/2014 | Spain | 2014 | *Sus_scrofa* |
| MT821905 | SeCoV-Italy/77590/2019 | Italy | 2019 | *Sus_scrofa* |
| **Genbank a.n.** | **isolate name** | **sampling location** | **sampling year** | **host species** |
| MT985156 | PDCoV_SCCZ18 | China | 2018 | *Sus_scrofa* |
| MT985157 | PDCoV_SCCZ18-10 | China | 2018 | *Sus_scrofa* |
| MT985158 | PDCoV_SCCZ18-73 | China | 2019 | *Sus_scrofa* |
| MT985160 | PDCoV_SCCZ18-140 | China | 2020 | *Sus_scrofa* |
| MT985161 | PDCoV_SCCZ18-160 | China | 2020 | *Sus_scrofa* |
| MW165134 | PHEV_67N/US/1970 | USA | 1970 | *Sus_scrofa* |
| MW165327 | PEDV_5-17-O | Taiwan | 2014 | *Sus_scrofa* |
| MW165328 | PEDV_5-17-V | Taiwan | 2014 | *Sus_scrofa* |
| MW165329 | PEDV_4-2 | Taiwan | 2013 | *Sus_scrofa* |
| MW413556 | PEDV_HB/HEBEU/2020 | China | 2020 | *Sus_scrofa* |
| MW560715 | PEDV_KNU-1907 | South Korea | 2019 | *Sus_scrofa* |
| MW804449 | TGEV_CH8438 | China | 2017 | *Sus_scrofa* |
| MW805354 | PEDV_DTI1 | Thailand | 2019 | *Sus_scrofa* |
| MW805355 | PEDV_DTI2 | Thailand | 2019 | *Sus_scrofa* |
| MW805356 | PEDV_DTI3 | Thailand | 2019 | *Sus_scrofa* |
| MZ268115 | PEDV_25364/2 | Poland | 2015 | *Sus_scrofa* |
| MZ291567 | PDCoV/OH-FD22 P7 | USA | 2014 | *Sus_scrofa* |
| MZ313556 | PEDV_0100/4T | Poland | 2017 | *Sus_scrofa* |
| MZ313557 | PEDV_6220 | Poland | 2016 | *Sus_scrofa* |
| MZ322950 | TGEV_CH/GX/TGEV/2662/2019 | China | 2019 | *Sus_scrofa* |
| MZ364307 | PEDV_CH/GX/PEDV/938/2016 | China | 2016 | *Sus_scrofa* |
| MZ364311 | PEDV_CH/GX/PEDV/1902/2017 | China | 2017 | *Sus_scrofa* |
| MZ364312 | PEDV_CH/GX/PEDV/1939/2018 | China | 2018 | *Sus_scrofa* |
| MZ368889 | TGEV_HB-1 | China | 2020 | *Sus_scrofa* |
| MZ388469 | PDCoV_CH/GX/PDCoV/1423/2016 | China | 2016 | *Sus_scrofa* |
| MZ388470 | PDCoV_CH/GX/PDCoV/1472A/2017 | China | 2017 | *Sus_scrofa* |
| MZ388472 | PDCoV_CH/GX/PDCoV/1539C/2017 | China | 2017 | *Sus_scrofa* |
| MZ772936 | PDCoV_BN | China | 2016 | *Sus_scrofa* |
| **Genbank a.n.** | **isolate name** | **sampling location** | **sampling year** | **host species** |
| MZ787936 | PEDV_TQ_VN | Vietnam | 2018 | *Sus_scrofa* |
| MZ787937 | PEDV_HN_VN | Vietnam | 2018 | *Sus_scrofa* |
| MZ803010 | PEDV_UG/Canada/Ontario/2014 | Canada | 2014 | *Sus_scrofa* |
| OK078017 | SADS_CH-JX2/2018 | China | 2018 | *Sus_scrofa* |
| OK078898 | PRCV-1/90-DK | Denmark | 1990 | *Sus_scrofa* |
| OL348059 | PEDV_XJ1904-34 | China | 2019 | *Sus_scrofa* |
| OL542832 | PHEV_GNU-2113 | South Korea | 2021 | *Sus_scrofa* |
| OM047182 | PDCoV_CHN-SCMY2021-01 | China | 2021 | *Sus_scrofa* |
| OM777140 | PDCoV_NTU/C253/21 | Taiwan | 2021 | *Sus_scrofa* |
| OM802899 | TGEV_SZ19 | China | 2019 | *Sus_scrofa* |
| OM830318 | PRCV_86/135308 | UK | 1986 | *Sus_scrofa* |
| OM830319 | PRCV_310 isolate AR310 | USA | 1993 | *Sus_scrofa* |
| OM830320 | PRCV_86/135308 | UK | 1986 | *Sus_scrofa* |
| OM830321 | PRCV_ISU-1 | USA | 1990 | *Sus_scrofa* |
| ON858825 | TGEV_SC2021 | China | 2021 | *Sus_scrofa* |
| ON859974 | TGEV_HNSQ-2021 | China | 2021 | *Sus_scrofa* |
| ON911569 | SADS_Guangxi/2021 | China | 2021 | *Sus_scrofa* |
| ON936274 | PDCoV_CZ2020-F80 | China | 2020 | *Sus_scrofa* |
| OP434397 | TGEV_HN-2012 | China | 2012 | *Sus_scrofa* |
| OP566509 | PDCoV_YRQ/2016 | China | 2016 | *Sus_scrofa* |
| OP805351 | TGEV_DS01-2022 | China | 2022 | *Sus_scrofa* |
| OP959790 | PHEV_LJ/2021 | China | 2021 | *Sus_scrofa* |
| OP972835 | PEDV_YNBS/2022 | China | 2022 | *Sus_scrofa* |
| OQ305205 | PHEV_GD/2017 | China | 2017 | *Sus_scrofa* |
| OQ305206 | PHEV_HLJ/2017 | China | 2017 | *Sus_scrofa* |
| OQ305207 | PHEV_PHEV/SC/2017 | China | 2017 | *Sus_scrofa* |
| OQ305208 | PHEV_PHEV/ZJ/2017 | China | 2017 | *Sus_scrofa* |
| OQ349200 | PEDV_SD1-2021 | China | 2021 | *Sus_scrofa* |
| OQ437174 | PEDV_YNLP/2022 | China | 2022 | *Sus_scrofa* |
| OQ437175 | PEDV_YN2021 | China | 2021 | *Sus_scrofa* |
| **Genbank a.n.** | **isolate name** | **sampling location** | **sampling year** | **host species** |
| OQ473581 | PDCoV_JS2021-LX | China | 2022 | *Sus_scrofa* |
| OQ547740 | PDCoV_GX2021-1 | China | 2021 | *Sus_scrofa* |
| OQ736716 | PDCoV_GX2022-2 | China | 2022 | *Sus_scrofa* |
| OQ790129 | PDCoV_SN-Swine-2018 | China | 2018 | *Sus_scrofa* |
| OQ798806 | PHEV_rvPHEV17 | China | 2018 | *Sus_scrofa* |
| OQ798807 | PHEV_rvPHEV1 | China | 2021 | *Sus_scrofa* |
| OQ798808 | PHEV_rvPHEV10 | China | 2021 | *Sus_scrofa* |
| OQ798809 | PHEV_rvPHEV13 | China | 2021 | *Sus_scrofa* |
| OQ798810 | PHEV_rvPHEV14 | China | 2019 | *Sus_scrofa* |
| OQ798811 | PHEV_rvPHEV15 | China | 2019 | *Sus_scrofa* |
| OQ798812 | PHEV_rvPHEV16 | China | 2020 | *Sus_scrofa* |
| OQ798813 | PHEV_rvPHEV18 | China | 2021 | *Sus_scrofa* |
| OQ798814 | PHEV_rvPHEV19 | China | 2021 | *Sus_scrofa* |
| OQ798815 | PHEV_rvPHEV2 | China | 2021 | *Sus_scrofa* |
| OQ798816 | PHEV_rvPHEV20 | China | 2021 | *Sus_scrofa* |
| OQ798817 | PHEV_rvPHEV21 | China | 2020 | *Sus_scrofa* |
| OQ798818 | PHEV_rvPHEV22 | China | 2021 | *Sus_scrofa* |
| OQ798819 | PHEV_rvPHEV23 | China | 2021 | *Sus_scrofa* |
| OQ798820 | PHEV_rvPHEV24 | China | 2021 | *Sus_scrofa* |
| OQ798821 | PHEV_rvPHEV25 | China | 2021 | *Sus_scrofa* |
| OQ798822 | PHEV_rvPHEV26 | China | 2021 | *Sus_scrofa* |
| OQ798823 | PHEV_rvPHEV3 | China | 2021 | *Sus_scrofa* |
| OQ798824 | PHEV_rvPHEV4 | China | 2019 | *Sus_scrofa* |
| OQ798825 | PHEV_rvPHEV5 | China | 2016 | *Sus_scrofa* |
| OQ798826 | PHEV_rvPHEV6 | China | 2020 | *Sus_scrofa* |
| OQ798827 | PHEV_rvPHEV7 | China | 2019 | *Sus_scrofa* |
| OQ798828 | PHEV_rvPHEV8 | China | 2020 | *Sus_scrofa* |
| OQ798829 | PHEV_rvPHEV9 | China | 2019 | *Sus_scrofa* |
| OQ915150 | PEDV_ZJ2022 | China | 2022 | *Sus_scrofa* |
| OQ979200 | PEDV_HNZK1 | China | 2021 | *Sus_scrofa* |
| **Genbank a.n.** | **isolate name** | **sampling location** | **sampling year** | **host species** |
| OR122653 | PDCoV_HNZK-02-P120 | China | 2021 | *Sus_scrofa* |
| OR209251 | PRCV_USA/AR310/1989_ISU | USA | 1989 | *Sus_scrofa* |
| OR209252 | PRCV_USA/LEPP1/1991_ISU | USA | 1991 | *Sus_scrofa* |
| OR209253 | PRCV_USA/1894X/1992_ISU | USA | 1992 | *Sus_scrofa* |
| OR209254 | PRCV_USA/ISU20-92330/2020 | USA | 2020 | *Sus_scrofa* |
| OR230676 | PDCoV_HeN17 | China | 2017 | *Sus_scrofa* |
| OR689863 | PRCV_15087/12 III NPTV Parma | Italy | 2012 | *Sus_scrofa* |
| OR689864 | PRCV_91V44 | Belgium | 1991 | *Sus_scrofa* |
| OR762053 | PDCoV_ZD2022 | China | 2022 | *Sus_scrofa* |
| PP069800 | SADS_HNNY/2023 | China | 2023 | *Sus_scrofa* |
| PP236916 | TGEV_VET-16 | Vietnam | 2016 | *Sus_scrofa* |
| PP441972 | PEDV_GNU-2389 | South Korea | 2023 | *Sus_scrofa* |
| PP441974 | PEDV_GNU-2401 | South Korea | 2024 | *Sus_scrofa* |
| PP998011 | PDCoV_GX2024-2 | China | 2024 | *Sus_scrofa* |
| PP999774 | PEDV_LZ202401 | China | 2024 | *Sus_scrofa* |
| PQ000331 | SADS_GD-1 | China | 2022 | *Sus_scrofa* |
| PQ141088 | PEDV_OH-24-5439 | USA | 2024 | *Sus_scrofa* |
| PQ181669 | PEDV_C17 | Vietnam | 2015 | *Sus_scrofa* |
| PQ189446 | TGEV_Ly23 | China | 2023 | *Sus_scrofa* |
| PQ241464 | PHEV_USA/IL/19661/2021 | USA | 2021 | *Sus_scrofa* |
| PQ316091 | PEDV_CH/HBJL2023 | China | 2023 | *Sus_scrofa* |
| PQ316092 | PEDV_CH/ShXXY2-2023 | China | 2023 | *Sus_scrofa* |
| PQ373831 | PDCoV_CHN/SX-Y/2023 | China | 2023 | *Sus_scrofa* |
| PQ539659 | PDCoV_CC23 | China | 2023 | *Sus_scrofa* |
| PQ645844 | PDCoV_HZYH-2019 | China | 2019 | *Sus_scrofa* |
| PQ753145 | PDCoV_HuN/2023 | China | 2023 | *Sus_scrofa* |
