# Supplementary material for: A multi-disciplinary approach to identify spillover interfaces of bat coronaviruses to pig farms in Italy
Source: PLoS One. 2025 Oct 15;20(10):e0332117. doi: 10.1371/journal.pone.0332117 (PMC12527140; doi:10.1371/journal.pone.0332117)

**Fig S1. Ternary plots of predicted overall activity (top row), *P. kuhlii* activity and richness as a function of land-use (proportion of urban, agriculture or wood landscape in the buffer)**.

Mean, lower and upper 95%CI predictions are shown in the left, middle and right column respectively. Black dots represent land-use in the dataset.


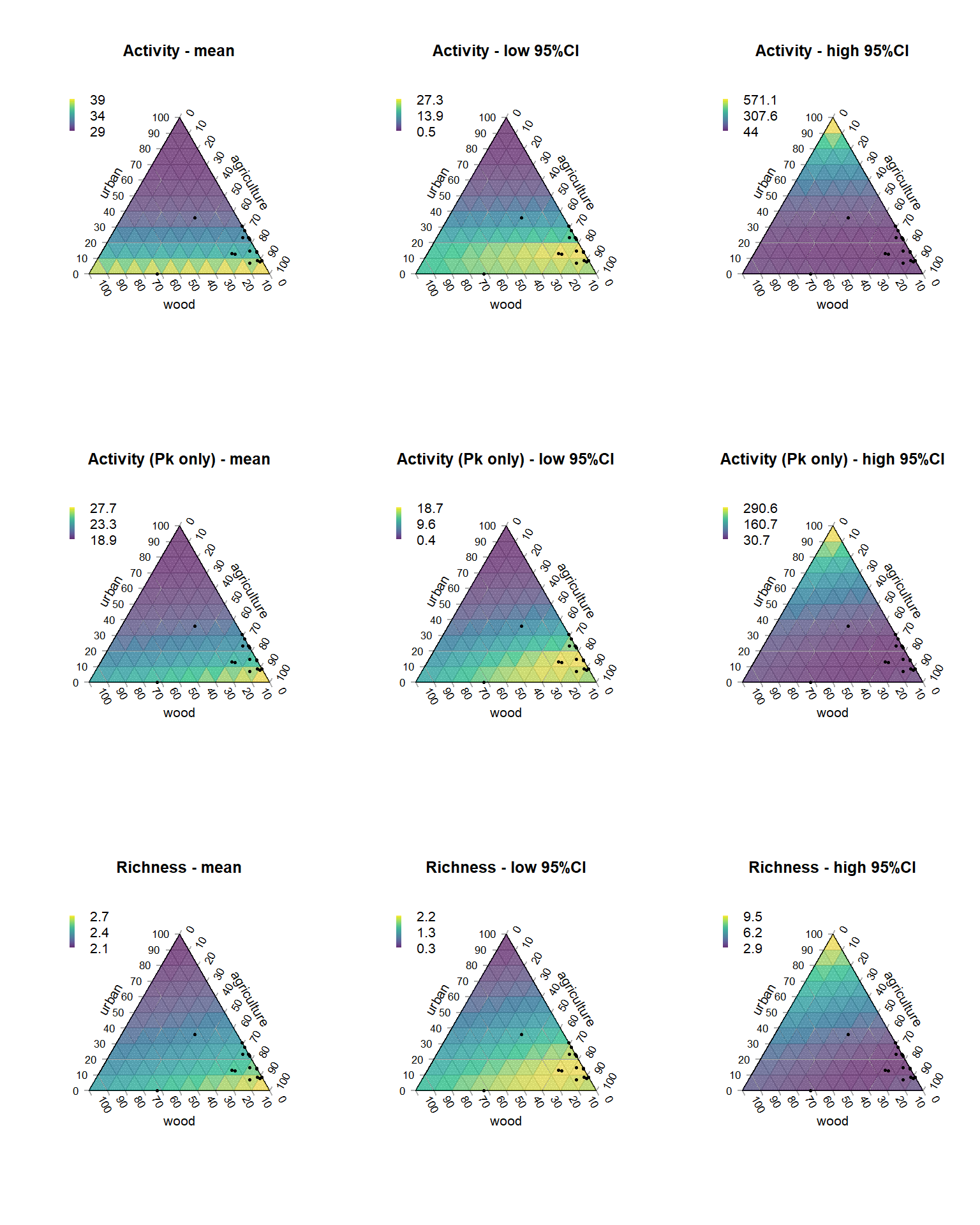

Supplement: S1 Fig — (DOCX) [file pone.0332117.s010.docx]
