# Supplementary material for: A multi-disciplinary approach to identify spillover interfaces of bat coronaviruses to pig farms in Italy
Source: PLoS One. 2025 Oct 15;20(10):e0332117. doi: 10.1371/journal.pone.0332117 (PMC12527140; doi:10.1371/journal.pone.0332117)

**Fig S2 (1 of 4). Relationship between observed (black) and predicted activity (left column), *P. kuhlii* activity (central column) and richness (right column), and each predictor variable in the dataset (rows).**

Predictions are based on multimodel averaging using up to 2 (red), 3 (blue), or 4 (green) predictor variables (see methods). No predictions are shown when the predictor was excluded based on univariate regression.

When the predictor is categorical: black dots/bars represent medians/ 10% and 90% percentiles of the observations; coloured dots/bars represent means and 95%CI predictions.

When the predictor is continuous: black dots represent the observations; coloured lines/shadings represent means and 95%CI predictions.


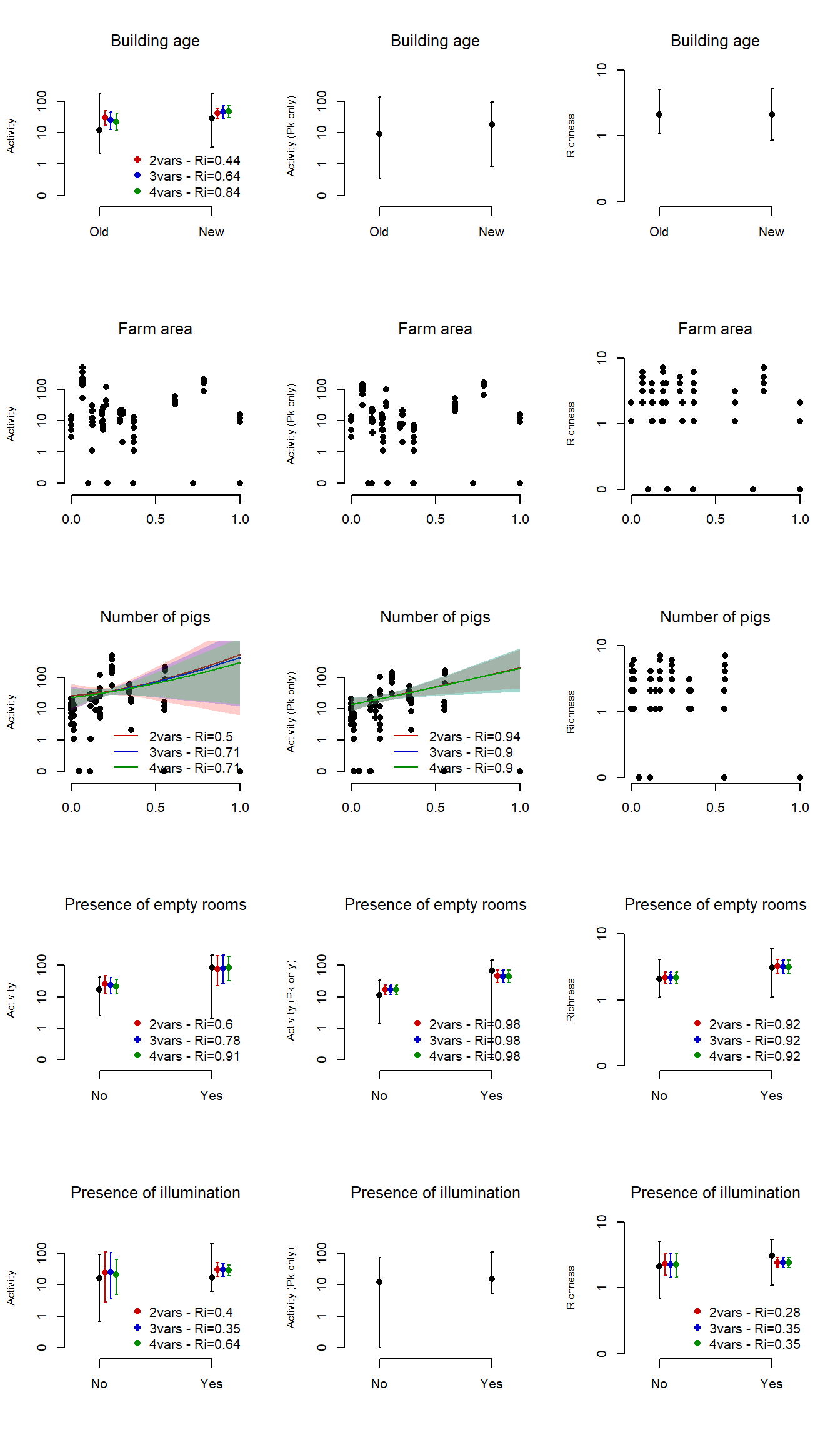


Fig S2 (2 of 4). Continued.


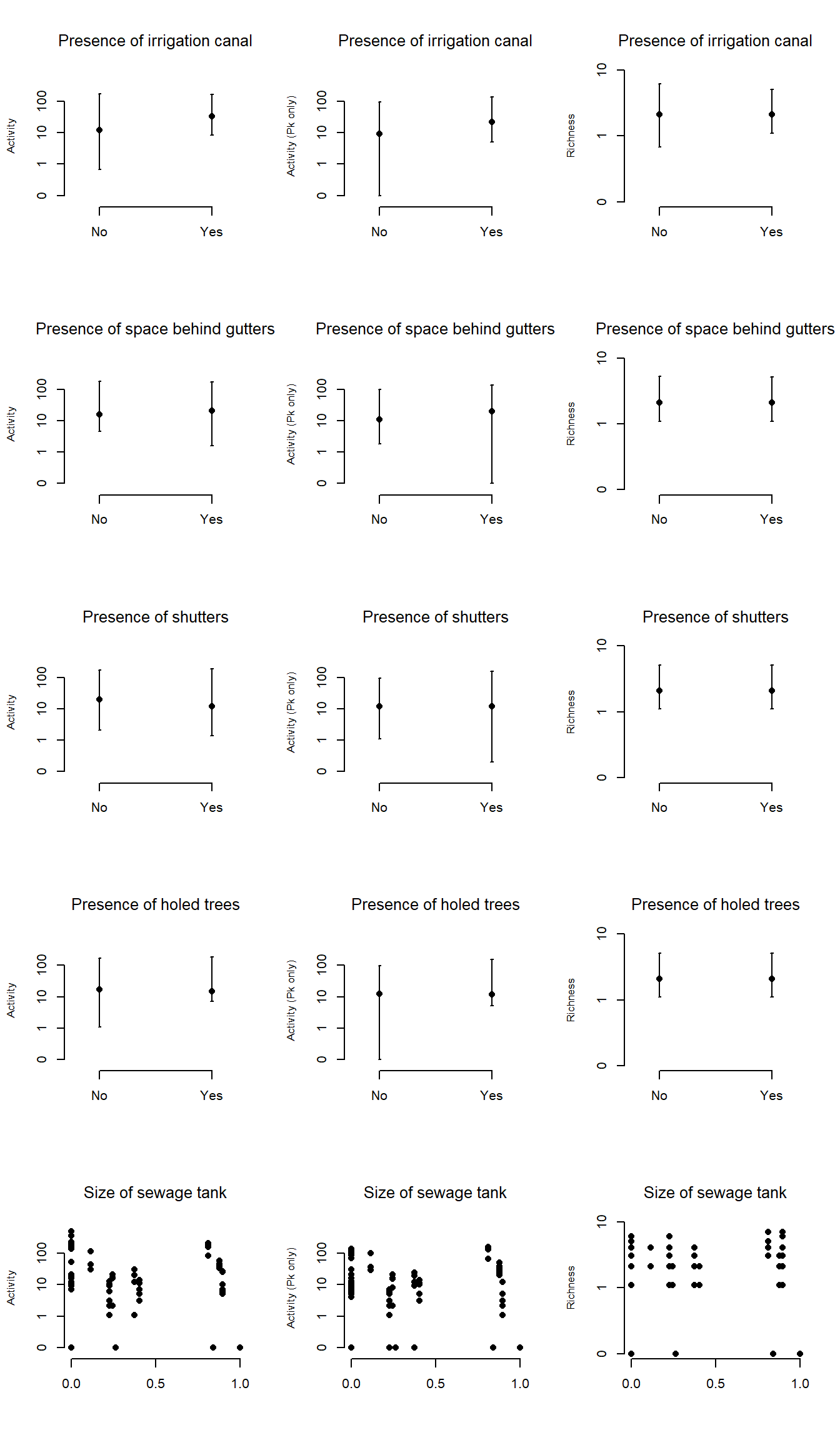


Fig S2 (3 of 4). Continued.


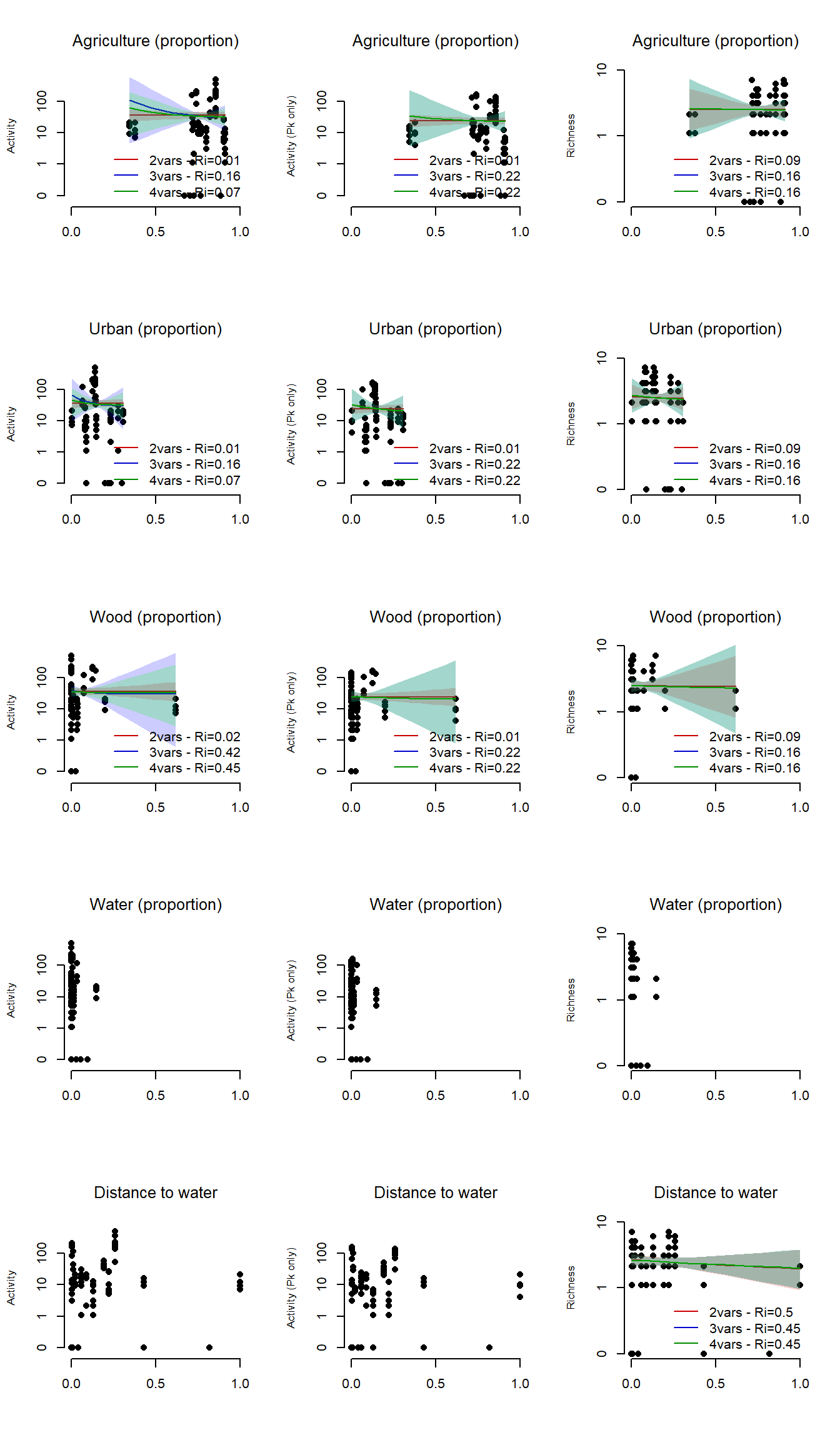


Fig S2. (4 of 4). Continued.


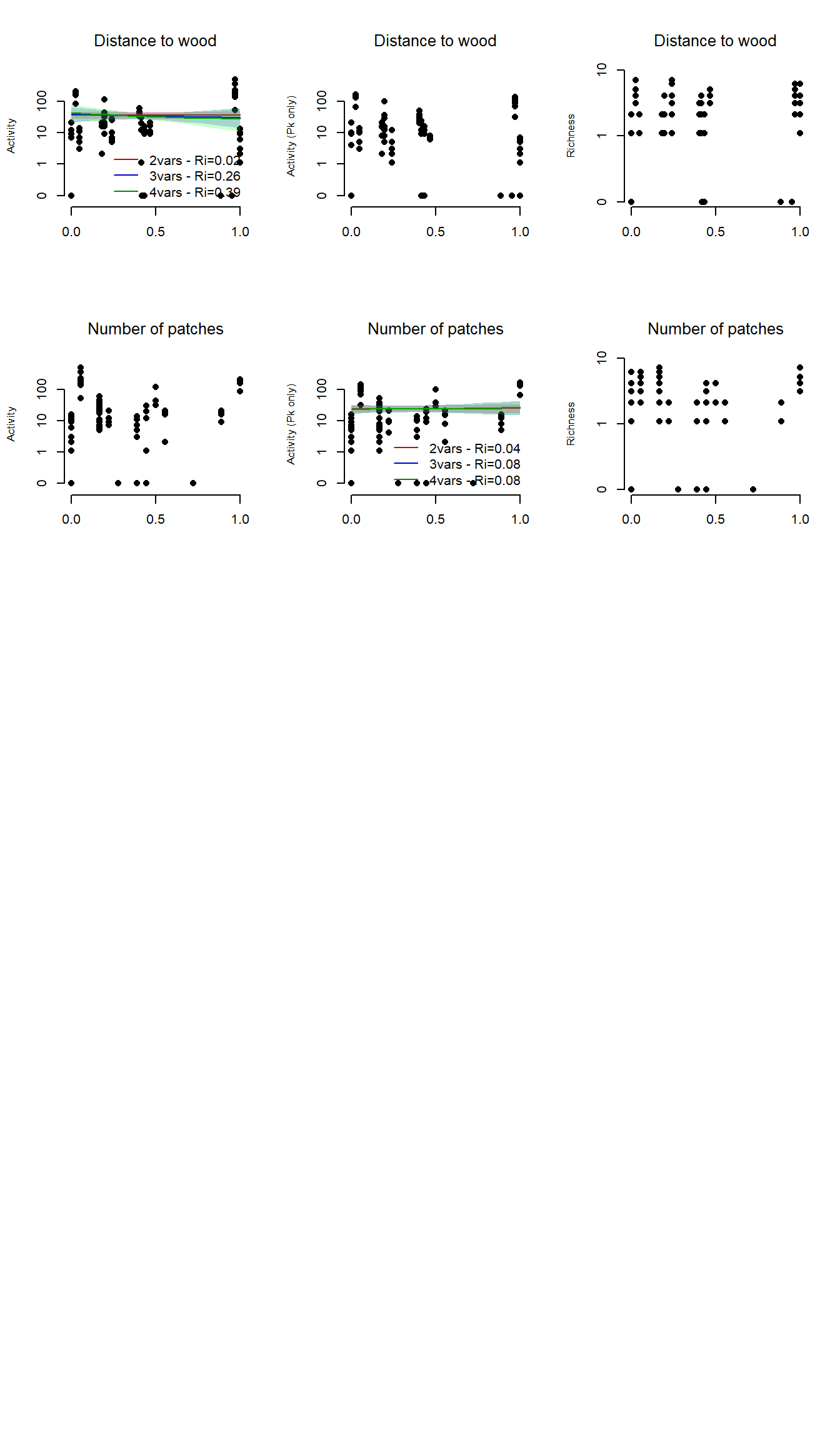

Supplement: S2 Fig — (DOCX) [file pone.0332117.s011.docx]
