# Supplementary material for: A multi-disciplinary approach to identify spillover interfaces of bat coronaviruses to pig farms in Italy
Source: PLoS One. 2025 Oct 15;20(10):e0332117. doi: 10.1371/journal.pone.0332117 (PMC12527140; doi:10.1371/journal.pone.0332117)

**Fig S3. Annotated structure of the novel genome of BtCoV_020.**

This structure was obtained using Geneious version 2022.1 created by Biomatters. Available from <https://www.geneious.com>


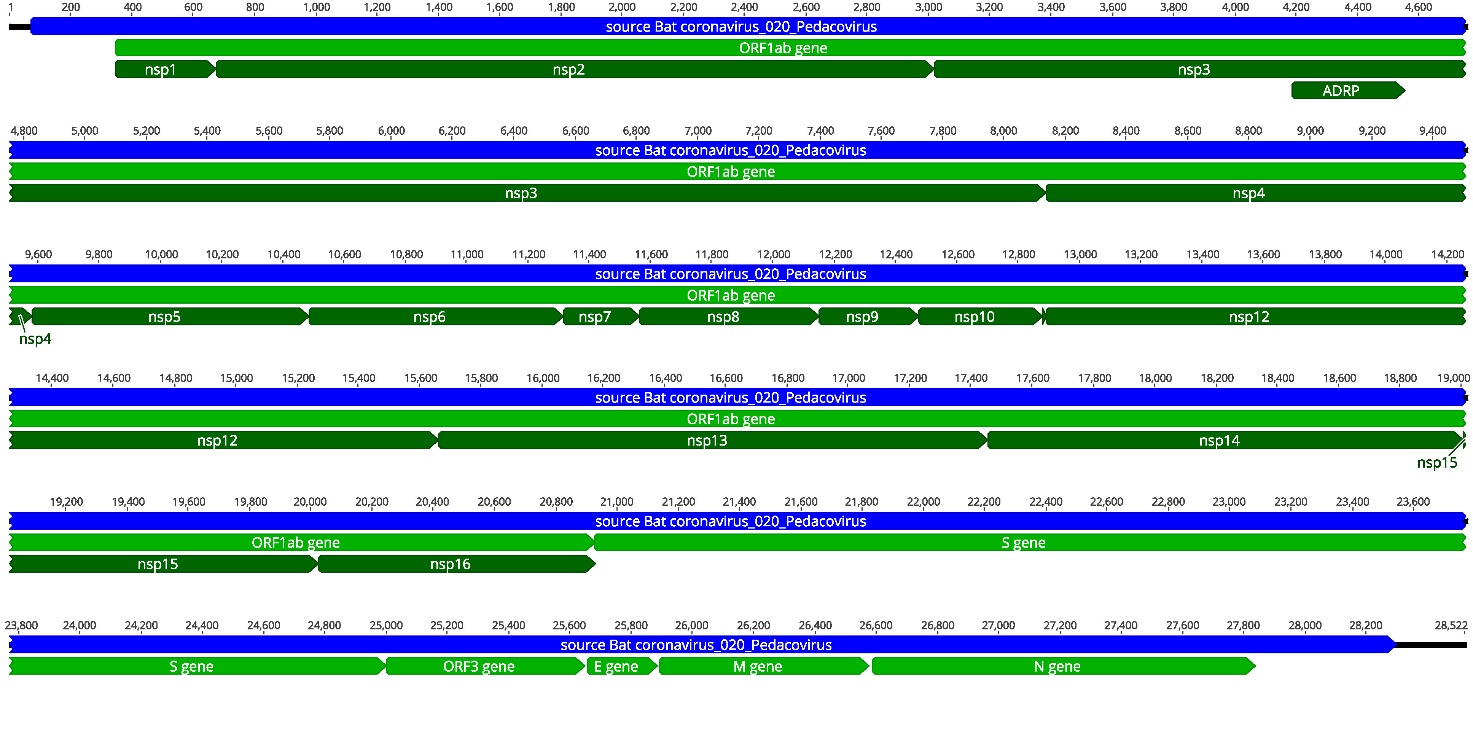

Supplement: S3 Fig — (DOCX) [file pone.0332117.s012.docx]
