# Supplementary material for: A multi-disciplinary approach to identify spillover interfaces of bat coronaviruses to pig farms in Italy
Source: PLoS One. 2025 Oct 15;20(10):e0332117. doi: 10.1371/journal.pone.0332117 (PMC12527140; doi:10.1371/journal.pone.0332117)
Supplement: S4 Fig — (DOCX) [file pone.0332117.s013.docx]

**Fig S4. Phylogenetic trees showing correlation of a novel strain of Italian BtCoV_020 with reference sequences, based on 8 ORF1ab structural proteins, ORF3, S, E, M and N proteins.**

Trees have been edited using iTOL, showing our sequence in red and three most similar sequences in blue, orange and green using colour strip.
