# Supplementary material for: A multi-disciplinary approach to identify spillover interfaces of bat coronaviruses to pig farms in Italy
Source: PLoS One. 2025 Oct 15;20(10):e0332117. doi: 10.1371/journal.pone.0332117 (PMC12527140; doi:10.1371/journal.pone.0332117)
Supplement: Attachment — Submitted filename: Response to Reviewers.docx [file pone.0332117.s015.docx]

*We thank editors and reviewers for their valuable comments and the appreciation of our work. We therefore submit a revised version where all comments have been addressed.*

Journal Requirements:

1. Please ensure that your manuscript meets PLOS ONE's style requirements, including those for file naming.

*New files were uploaded with corrected names. The style has been revised throughout the text*

2. To comply with PLOS One submissions requirements, in your Methods section, please provide additional information regarding the experiments involving animals and ensure you have included details on (1) methods of sacrifice, (2) methods of anesthesia and/or analgesia, and (3) efforts to alleviate suffering.

*Thank you for this comment. We now specified that no anesthesia has been performed during active surveillance, while applying best practices to avoid suffering and minimize disturbance. On the other hand, no animals have been sacrificed as all individuals included in passive surveillance are submitted as carcasses to the laboratory.*

3. Your ethics statement should only appear in the Methods section of your manuscript. If your ethics statement is written in any section besides the Methods, please delete it from any other section.

*Done*

4. When completing the data availability statement of the submission form, you indicated that you will make your data available on acceptance. We strongly recommend all authors decide on a data sharing plan before acceptance, as the process can be lengthy and hold up publication timelines. Please note that, though access restrictions are acceptable now, your entire data will need to be made freely accessible if your manuscript is accepted for publication. This policy applies to all data except where public deposition would breach compliance with the protocol approved by your research ethics board. If you are unable to adhere to our open data policy, please kindly revise your statement to explain your reasoning and we will seek the editor's input on an exemption. Please be assured that, once you have provided your new statement, the assessment of your exemption will not hold up the peer review process.

*As we stated how we deposited sequences in GenBank and SRA, we cannot fully understand this comment. This might be related with the temporary embargo on Genbank until the release of the paper that is common practice. However, we set final deadline for the embargo at September 1^st^ 2026 after which sequences will be released anyway.*

5. We note that the grant information you provided in the ‘Funding Information’ and ‘Financial Disclosure’ sections do not match.
   When you resubmit, please ensure that you provide the correct grant numbers for the awards you received for your study in the ‘Funding Information’ section.

*Thank you for this comment, we will check accordingly during the submission phases.*

6. Thank you for stating the following financial disclosure:
   The present work was supported by the First International ICRAD call under grant agreement N◦ 862605, ID 95 ConVErgence.

Please state what role the funders took in the study. If the funders had no role, please state: "The funders had no role in study design, data collection and analysis, decision to publish, or preparation of the manuscript."
If this statement is not correct you must amend it as needed.
Please include this amended Role of Funder statement in your cover letter; we will change the online submission form on your behalf.

*Done*

7. [We note that Figure 1 in your submission contain [map/satellite] images which may be copyrighted. All PLOS content is published under the Creative Commons Attribution License (CC BY 4.0), which means that the manuscript, images, and Supporting Information files will be freely available online, and any third party is permitted to access, download, copy, distribute, and use these materials in any way, even commercially, with proper attribution. For these reasons, we cannot publish previously copyrighted maps or satellite images created using proprietary data, such as Google software (Google Maps, Street View, and Earth). For more information, see our copyright guidelines: http://journals.plos.org/plosone/s/licenses-and-copyright.

   We require you to either (1) present written permission from the copyright holder to publish these figures specifically under the CC BY 4.0 license, or (2) remove the figures from your submission:](We%20note%20that%20Figure%201%20in%20your%20submission%20contain%20%5bmap/satellite%5d%20images%20which%20may%20be%20copyrighted.%20All%20PLOS%20content%20is%20published%20under%20the%20Creative%20Commons%20Attribution%20License%20(CC%20BY%204.0),%20which%20means%20that%20the%20manuscript,%20images,%20and%20Supporting%20Information%20files%20will%20be%20freely%20available%20online,%20and%20any%20third%20party%20is%20permitted%20to%20access,%20download,%20copy,%20distribute,%20and%20use%20these%20materials%20in%20any%20way,%20even%20commercially,%20with%20proper%20attribution.%20For%20these%20reasons,%20we%20cannot%20publish%20previously%20copyrighted%20maps%20or%20satellite%20images%20created%20using%20proprietary%20data,%20such%20as%20Google%20software%20(Google%20Maps,%20Street%20View,%20and%20Earth).%20For%20more%20information,%20see%20our%20copyright%20guidelines:%20http://journals.plos.org/plosone/s/licenses-and-copyright.We%20require%20you%20to%20either%20(1)%20present%20written%20permission%20from%20the%20copyright%20holder%20to%20publish%20these%20figures%20specifically%20under%20the%20CC%20BY%204.0%20license,%20or%20(2)%20remove%20the%20figures%20from%20your%20submission:a.%20You%20may%20seek%20permission%20from%20the%20original%20copyright%20holder%20of%20Figure%201%20to%20publish%20the%20content%20specifically%20under%20the%20CC%20BY%204.0%20license.We%20recommend%20that%20you%20contact%20the%20original%20copyright%20holder%20with%20the%20Content%20Permission%20Form%20(http://journals.plos.org/plosone/s/file?id=7c09/content-permission-form.pdf)%20and%20the%20following%20text:)

*Thank you for this comment. We actually did not access to copyrighted information while using the licenced software ArcGIS Pro that should hold all permission for their users. We now changed the figure caption and deleted the source within the figure. Would this be fine for you?*

8. [Please remove all personal information, ensure that the data shared are in accordance with participant consent, and re-upload a fully anonymized data set.
   Note: spreadsheet columns with personal information must be removed and not hidden as all hidden columns will appear in the published file.
   Additional guidance on preparing raw data for publication can be found in our Data Policy (https://journals.plos.org/plosone/s/data-availability#loc-human-research-participant-data-and-other-sensitive-data) and in the following article: http://www.bmj.com/content/340/bmj.c181.long.](Please%20remove%20all%20personal%20information,%20ensure%20that%20the%20data%20shared%20are%20in%20accordance%20with%20participant%20consent,%20and%20re-upload%20a%20fully%20anonymized%20data%20set.Note:%20spreadsheet%20columns%20with%20personal%20information%20must%20be%20removed%20and%20not%20hidden%20as%20all%20hidden%20columns%20will%20appear%20in%20the%20published%20file.Additional%20guidance%20on%20preparing%20raw%20data%20for%20publication%20can%20be%20found%20in%20our%20Data%20Policy%20(https://journals.plos.org/plosone/s/data-availability#loc-human-research-participant-data-and-other-sensitive-data) and in the following article: http://www.bmj.com/content/340/bmj.c181.long.)

*We agree that a fully anonymized dataset is key to guarantee privacy. The questionnaire responded to international regulation and has been submitted by farm owners. In addition, no personal data are shown in the study, where farms have been randomly labelled 1 to 14 so that no identification of exact sites is possible. We now specified this in the method section. While we double checked that no information have been mistakenly provided, we ask you to highlight any error from our side so that we can amend it.*

9. If the reviewer comments include a recommendation to cite specific previously published works, please review and evaluate these publications to determine whether they are relevant and should be cited. There is no requirement to cite these works unless the editor has indicated otherwise.

*Done*

Reviewers' comments to the Author

Reviewer #1: Authors analysed the bat-swine interface, to highlight a potential transmission route of bat coronaviruses to swine, and some related risk factors. The increasing relevance of swine farming in the food chain and the risks for pathogens spillovers from wildlife to swine, support the need for such studies. Under this light, this study contributes with some interesting still little explored information to aware of potential risks to pig farms. This study follows a previous one (doi: 10.3390/v13010004), based on more limited data, improving the data and, therefore, it appears of interest.

*Thank you for your appreciation*

In some parts it tentatively tried to describe the potential spillover of bat coronaviruses to pigs, even if overall it does not include data on these viruses in pigs, despite the potentially described chances of interaction between species.

*While we actually sampled swine, no bat viruses have been found and the work was devolved to a study of swine coronaviruses that is in preparation. We now acknowledge this study in both the results and the discussion but unfortunately we have no DOI yet to reference to this.*

Overall, it appears well written and methodologically supported, even if some parts could benefit of synthesis for more clarity. I below added some additional comments and suggestions for the Authors.

*Thank you for your appreciation of our work.*

Major comments:
- I suggest defining the names of bat species in italics.

*Done*

- Line 94: To avoid unnecessary misinterpretations, I suggest clarifying if these two cited novel genera are taxonomically officially recognized or tentatively defined with these proposed genera.

*Done*

- Lines 126-128: I suggest briefly including more details on the investigated pig farms, to be more descriptive with such a wide audience of readers, not limited to this specific geographical context.

*Thank you for this comment. We revised the text for more description about the pig farms considered.*

- Line 131: Did Authors consider biosafety measures generically to influenza viruses or specifically only to H5N1 avian influenza virus? In any case, I suggest specifying also the virus name rather than the only viral genotype.

*Thank you for your comments. The sentence has been revised for more clarity to highlight how during H5N1 (avian flu) epidemics access to swine farms was highly limited, while being completely blocked after the first case of ASF. This justified why we could not sample more, unfortunately.*

- Lines 177-178: Did Authors concretely observe the contamination with bat feces inside the pig housing areas or demonstrate/hypothesize a way of introduction? It doesn’t appear clear, as it was described.

*Thank you for this comment. We revised the text for more clarity. Indeed, we used open windows and lack of grids as risk factors allowing entrance of bats or faeces but we could not standardize the collection of information on the presence/absence of bats or faeces.*

- Lines 240-241: “frequently found”, as currently expressed, is not referred to this specific farming context, as well “more active” is not referred to, therefore I suggest clarifying this sentence. Moreover, is this reference referred to “all study sites”? Did this reference include information on the common observed bats close to pig farms in Italy, as reported in other studies cited below? This should be clarified, also to support the text at lines 284-285. Finally, was the “pooled faecal material” (cited at line 242) tested for the species of origin (as described at line 292)?

*Thank you for this comment. We revised the text for more clarity. These data refer to actual results coming from the ecological part of the study, even if was also supported by previous investigation referenced. We also specified the performance of genetic ID on pooled environmental samples as suggested*

- Lines 371-372: As this sentence was expressed, it appears that the analysis was limited to those viruses that are documented to be responsible for interspecies transmission events. Is there any reference to support or is this a speculative hypothesis?

*Thank you for noticing possible misunderstanding of this statement. Indeed, we consider that spillover is possible only for viruses that are actually present and we tried to express this concept, even if we realize that this is actually speculative as we might overlook unknown viruses. The sentence has been deleted to avoid misunderstanding.*

- Lines 616-617: this sentence is not clear enough: how can this data be beneficial for risk of the introduction of other (not bat-related) viruses in pig farms?

*Thank you for this comment. We revised the text for more clarity.*

- Lines 666 and 720: Did this study evaluate CoVs in bats in close contact to swine farms or directly bat CoVs “in” swine farms? Did this study investigate spillovers of viruses from bats to pigs?

*Thank you for this comment. We revised the text to distinguish the aim addressed in this study as to investigate the presence of bat coronaviruses in bats circulating at the interface with pigs in Italian pig farms. While we investigated pigs as well data are presented in another study on swine coronaviruses, while no bat covs were found*

- Line 724: As this study is interesting, further efforts should be made probably also towards other types of pig farming, as rural and semi-extensive farming are widely diffuse, not only to Italy but also in other parts of the globe, with closer and less controlled human-pig contacts.

*Thank you for this comment. We agree that enlarging our sampling is interesting and is worthy for future funding. This was not possible in ConVergence project as IZSVe only has territorial competence for animal diseases in the study area.*

Minor comments:
- Line 91: I suggest using italics characters for “Nidovirales”.

*Done*

- Line 92: I suggest adding a comma before “and Deltacoronavirus..”

*Done*

- Line 267: what do “, 2020” and “for faeces” mean?

*Thank you for noticing this. It was a typo that has been corrected*

- Line 271: I suggest adding “titled” just after “paragraph”.

*Done*

- Lines 288-289: I suggest replacing “in Istituto” with “at Istituto”, and to add “,Italy” just after “Venezie”.

*Done*

- Line 381: Should “respectively” be included?

*Added*

- Lines 529-532: I suggest using italics characters for the subfamily names.

*Done*

- Line 557: I suggest including the related accession numbers.

*Thank you for the suggestion, done.*

- Lines 566 and 567: I suggest preferring “GenBank accession number” or “accession number” to “gb an”.

*Replaced throughout the text*

- Line 750: I suggest moving “Sanger” before “sequencing” or add “method” after “Sanger”.

*Done*

Reviewer #2: The authors explored the possible spillover of CoVs at the interface between bats and pigs in intensive farming systems in Northeastern Italy through a holistic and multidisciplinary approach, applying methodologies from ecology, virology, and epidemiology. This is an interesting study that employs a sound multidisciplinary analysis methodology. However, the study has a significant sampling issue.

The dates on which the acoustic sampling was carried out are not specified, and apparently, only one sampling per farm was conducted during the period between April and October. It must be taken into account that variability in bat activity and diversity changes significantly across different times of the year and even between sampling events within the same period. In this regard, sampling must be carefully planned, and multiple surveys should be conducted throughout the April to October period. The part of the discussion that talks about the observed bat activity and diversity should be reviewed. Furthermore, in order to reliably compare bat activity and species diversity across farms, sampling must be carried out within the same temporal window. It is not clear that the authors followed this approach.

*Thank you for the comments. Despite the full agreement on the variability across sites and sampling periods concerning the phenology of the species, the rates of activity and the richness in the population recorded, we had to address several complications (H5N1 and ASF epidemics) that impede us to perform replicates of sampling session in the same farm throughout the sampling year and between years. This represents the main limitation of our study and we clearly stated in the revised text to underline the need, in further similar analysis, to perform sound bioacustic monitoring projects.*

Additionally, the recording period appears to be too short. It is important to consider that some species emerge later at night and that others use human structures for temporary roosting during nocturnal activity, potentially after midnight.

*Thank you for the comments, and we agree completely on this point. Moreover, we decided to set the instruments with this temporal window to maximize the performances in terms of sampling days as much as possible. Concerning the fact that the noise of the piggeries would in any case trigger the recorders to switch on, we minimize the battery usage to the restricted time frame of sunset-midnight.* *Future direction will be of considering external batteries to be linked to acoustic devices in order to fully perform a systematic and sound acoustic monitoring*. *This issue has been addressed in the revised text.*

Nevertheless, the article provides valuable data, particularly regarding CoV circulation in P. kuhlii. I recommend that the authors explicitly acknowledge these sampling limitations and clearly state that the data presented are preliminary. Further studies will be necessary to complement and expand upon the findings reported in this work.

*Thank you for these general comments, revision have been addressed in the text.*

Specific Suggestions and Edits:
Page 2, Line 71: Please change water to humidity.

*Thank you for this comment, although we believe that humidity is not the word we wanted to insert as we referred to the availability of feeding grounds, drinking sites and roosts. We changed in the text accordingly*.

Page 2, Line 77: Please add the sentence:
“...can offer essential drinking opportunities and food resources for bats.”

*Done*

Page 2, Lines 77–79: Replace:
“In fact, bats are particularly vulnerable to dehydration: due to their unique morphology and physiology, they happen to lose substantial amounts of water through their body surfaces”
with:
“In fact, bats are particularly vulnerable to dehydration due to their high body surface area in relation to body volume, and the presence of bare wing membranes, which facilitate transpiration.”

*Done*

Page 5, Line 153: Please write sp. without italics.

*Done*

Page 12, Line 379: Please replace bat sounds with bat calls.

*Done*

Page 12, Line 388: Please write sp. without italics.

*Done*

Page 20, Line 621: Please clarify the phrase “Why attractive to insects?” – more context or explanation is needed.

*Thank you for the clarification and we revised accordingly*

Page 23, Line 722: Please delete the word crucial – it may be too strong or unnecessary in this context.

*Done*
